# Non‐Pharmacological Interventions for Cough in Patients With Lung Cancer: A Systematic Review and Meta‐Analysis

**DOI:** 10.1111/jocn.70289

**Published:** 2026-03-15

**Authors:** Mengyao Cao, Xueyan Cheng, Victor Ho‐fun Lee, Chia‐Chin Lin, Denise Shuk Ting Cheung

**Affiliations:** ^1^ Li Ka Shing Faculty of Medicine, School of Nursing University of Hong Kong Hong Kong China; ^2^ Department of Clinical Oncology, Li Ka Shing Faculty of Medicine University of Hong Kong Hong Kong China

**Keywords:** cough, lung cancer, meta‐analysis, non‐pharmacological intervention, RCT, systematic review

## Abstract

**Background:**

Cough, a prevalent and debilitating symptom of lung cancer, remains poorly managed. Accumulating evidence on non‐pharmacological interventions for lung cancer cough necessitates systematic evaluation to assess their efficacy.

**Aim:**

To synthesise evidence on non‐pharmacological interventions for managing cough in lung cancer patients.

**Design:**

A systematic review and meta‐analysis following the Preferred Reporting Items for Systematic reviews and Meta‐Analyses reporting guideline.

**Methods:**

Nine databases were searched from inception to December 2024 to identify randomised controlled trials. Study quality was appraised using the Revised Cochrane Risk‐of‐Bias Tool for Randomised Trials. Meta‐analyses were performed for quantitative synthesis, with sources of heterogeneity examined using meta‐regression and subgroup analyses.

**Results:**

Thirty‐eight studies representing 2995 lung cancer patients were identified. These studies investigated acupuncture therapy, moxibustion, pulmonary rehabilitation, self‐management intervention, physical exercises, psychoeducation support, mindfulness, and multicomponent interventions. Non‐pharmacological interventions showed positive effects on cough severity and cough‐related quality of life. Additional benefits were observed for expectoration, dyspnea, and general quality of life. Pulmonary rehabilitation showed a greater effect on cough severity than other non‐pharmacological interventions.

**Conclusion:**

Non‐pharmacological interventions are promising in improving cough, expectoration, dyspnea, and general quality of life among lung cancer patients. Pulmonary rehabilitation showed the most promising effect. Future research should adopt objective cough measures in addition to self‐reported measures.

**Implications for the Profession and/or Patient Care:**

Non‐pharmacological interventions demonstrated potential effects in relieving cough and additional benefits in improving expectoration, dyspnea, and general quality of life among lung cancer patients. Healthcare professionals may adopt pulmonary rehabilitation for cough and related symptoms in lung cancer patients.

**Impact:**

As the first meta‐analysis addressing non‐pharmacological interventions for lung cancer cough, this study provides evidence supporting their clinical efficacy for improving cough and associated symptoms among patients with lung cancer.

**Patient or Public Contribution:**

No patient or Public contribution.

**Registration:**

PROSPERO CRD42024588729.

## Summary

What Does This Paper Contribute to the Wider Global Clinical Community?

- This review identifies non‐pharmacological interventions used in managing cough in lung cancer and provides evidence supporting their clinical efficacy.
- Pulmonary rehabilitation appears to be the most promising intervention for reducing cough severity in patients with lung cancer, whereas acupuncture and moxibustion also show considerable effects.
- Further studies should consider adopting both subjective and objective cough measures and ascertain the efficacy of some forms of non‐pharmacological interventions.

## 1 Introduction

Cough is one of the most pronounced and persistent symptoms commonly experienced by lung cancer (LC) patients, substantially impacting their physical and emotional well‐being and quality of life (Oksholm et al. 2015; Zhou, Lei, et al. 2024). Over half of LC patients experienced cough and approximately 18%–36.4% had moderate or severe coughing symptoms (Harle et al. 2020; Li et al. 2023; Liao et al. 2022; Lou et al. 2017; Wang and Huang 2023; Yang et al. 2024). Cough in LC patients is associated with a range of symptoms including expectoration, dyspnea, itchy throat, dry mouth, nausea, vomiting, reflux, pain, sleep disturbance, fatigue and numbness (Harle et al. 2020; Harle et al. 2019; Lou et al. 2017; Maguire et al. 2014; Molassiotis et al. 2011; Zhou, Lei, et al. 2024). These symptoms can elicit negative emotions such as annoyance, worry, fear, embarrassment, anxiety and depression, ultimately resulting in decreased treatment compliance, diminished social life, impaired function ability, and reduced quality of life (Harle et al. 2019; Maguire et al. 2014; Molassiotis et al. 2011; Whisenant et al. 2020; Zhou, Lei, et al. 2024). Beyond that, cough may be a key barrier to postoperative rehabilitation (Sun et al. 2024), and it is linked to greater mortality and shortened survival in LC patients (Cui et al. 2024; Stavem et al. 2021; Wang et al. 2010). Moreover, cough has been suggested as the sentinel symptom (the symptom indicating the occurrence of a symptom cluster) within the respiratory symptom cluster in patients with LC (Ju et al. 2023; Ma et al. 2022), meaning that managing cough may simultaneously reduce the occurrence of the symptom cluster. Hence, effective cough management is imperative for LC survivors.

### 1.1 The Review

To date, no systematic reviews have focused on cough management in LC patients, a population whose cough was reported as worse in terms of frequency and severity than that in patients with asthma and chronic obstructive pulmonary disease (Harle et al. 2019). In 2017, based on 17 trials of primarily low‐quality evidence, a guideline summarised evidence for cough management among LC patients, suggesting that certain non‐pharmacological (cough suppression) and pharmacological (demulcents, opioids, peripherally acting antitussives, or local anaesthetics) treatments, as well as endobronchial brachytherapy, might be useful (Molassiotis et al. 2017). Following the publishing of this guideline, observational research has increasingly demonstrated the potential of non‐pharmacological interventions (NPIs), such as pulmonary rehabilitation and acupuncture, in managing LC cough with minimal safety concerns (Luo et al. 2024; Xie et al. 2019). More randomised controlled trials (RCTs) have also delved into specific NPIs (e.g., acupuncture and exercise) in the context of LC cough management (Molassiotis et al. 2021; Zhang et al. 2024). Despite the emerging evidence regarding non‐pharmacologic cough treatments for LC patients, no meta‐analysis has been performed.

### 1.2 Aims

This study aims to identify and evaluate the effectiveness of non‐pharmacological interventions for managing cough in patients with lung cancer.

## 2 Methods

### 2.1 Design

This systematic review and meta‐analysis was performed and reported in accordance with the Cochrane Handbook for Systematic Reviews of Interventions (Version 6.4) (Higgins et al. 2024) and the Preferred Reporting Items for Systematic reviews and Meta‐Analyses (PRISMA) reporting guideline (Page et al. 2021). The study was pre‐registered on the PROSPERO database under CRD42024588729.

### 2.2 Search Methods

A comprehensive literature search was carried out from inception through December 2024 across nine electronic databases, including seven English databases (PubMed, EMBASE, Allied and complementary Medicine Database, Cumulative Index to Nursing and Allied Health Literature Plus, Cochrane Library, Web of Science, and ProQuest) and two Chinese databases (China National Knowledge Infrastructure and Wanfang Data). No filters were applied. An additional search through citation lists and grey literature was performed to examine potential eligible studies. Covidence, an online software platform, was adopted for deduplication and study screening. The eligibility of the studies was evaluated by two reviewers independently, with any discrepancies resolved through a consensus process. The search strategy is in Table S1.

### 2.3 Eligibility Criteria

1. Study design: RCTs published in English or Chinese.
2. Participants: Adults (aged ≥ 18 years) diagnosed with LC, irrespective of the disease stage or the presence of comorbidities.
3. Intervention: NPIs, including but not restricted to exercise, education, music therapy, acupoint therapy, moxibustion, physiotherapy, pulmonary rehabilitation, behavioural therapy, and any combination of the above. Studies comparing NPIs combined with medicine/herbs were included if their control condition was medicine/herbs alone. Also excluded were studies evaluating treatments such as surgical procedures, radiation, and acupoint applications, and studies using interventions with indistinguishable components.
4. Comparison: No restrictions were set for the comparators.
5. Outcomes: Studies that reported either the occurrence, frequency, severity or distress of cough symptoms (i.e., cough‐related quality of life).

### 2.4 Quality Appraisal

Two reviewers independently appraised study quality using the revised Cochrane risk‐of‐bias tool for randomised trials (RoB 2) (Sterne et al. 2019). Risk of bias assessment was determined across five individual domains and overall, with ratings of low risk, some concerns, or high risk. The certainty of evidence was assessed using the Grading of Recommendations, Assessment, Development and Evaluations (GRADE) framework (*Grade Handbook* 2013), and graded as high, moderate, low, or very low.

### 2.5 Data Extraction

Data extraction was completed by the first reviewer, MC, and confirmed by the second reviewer, XC. Disagreements between the two reviewers were handled through a consensus process. The primary outcome of this review was cough, with secondary outcomes being expectoration, dyspnea, and general quality of life. Study information including general study characteristics (i.e., authors, year of publication, country), demographic characteristics of participants (i.e., age, sex, sample size and medical condition), type and duration of interventions, comparators, key outcome measures, adverse events, and attrition rates were extracted from the identified studies. In the event of any missing, unreported, or questionable data in eligible studies, we attempted to contact the study investigators through email. When studies assessed outcomes at multiple endpoints or using more than two measures, we prioritised the earliest post‐treatment data and the measure that was validated or the most used. For multi‐arm studies, we extracted data from groups where the only difference was the intervention investigated. All the extracted data were documented in an Excel spreadsheet.

### 2.6 Synthesis

Quantitative synthesis was conducted using R statistics software (Version 4.3.2) with post‐treatment mean and standard deviation (SD). Data were imputed when presented in other forms and adjusted when outcome measures pointed in the reverse direction. A meta‐analysis was conducted with a minimum of two homogenous studies. An overall effect size was estimated in the meta‐analysis with a standardised mean difference (SMD) using Hedges' *g* and a 95% confidence interval (CI). The effect size was deemed small, medium, or large if Hedges' *g* was 0.2–0.49, 0.5–0.79, or ≥ 0.8, respectively (Hedges and Olkin 2014). A narrative synthesis was provided when a quantitative synthesis was inapplicable.

Cochran's *Q* (*Chi* ^*2*^ statistic) and *I* ^*2*^ value were used to evaluate statistical heterogeneity across the included studies. A *Chi* ^*2*^ test with a *p*‐value smaller than 0.1 indicates a significant variation in effect estimates for individual studies, whereas a large *I* ^2^ (> 50%) reflects the overall between‐study variation (Deeks et al. 2023). When significant heterogeneity was indicated (*p* < 0.10 and *I* ^2^ > 50%), a random‐effects model was applied for the meta‐analysis (Deeks et al. 2023), and sources of heterogeneity were initially explored through univariate meta‐regression (when applicable) based on pre‐specified potential covariates, including country (China vs. other countries), participants (having cough at baseline vs. not necessarily having baseline cough), intervention type (acupuncture therapy, moxibustion, pulmonary rehabilitation, self‐management intervention, physical exercise, multicomponent intervention, and other types), comparator (contained Western and/or Chinese medicine vs. not contained Western and/or Chinese medicine), treatment duration (≤ 30 days vs. > 30 days) and instruments (validated vs. not validated), and further by subgroup analyses according to the covariates identified from the meta‐regression. A minimum of 10 studies in the meta‐analysis were required for the meta‐regression analysis (Deeks et al. 2023). When the meta‐regression was inapplicable, subgroup analyses were conducted based on the aforementioned potential covariates. Sensitivity analysis was conducted using leave‐one‐out cross‐validation through sequentially removing each study from the pooling. If more than 10 studies were pooled for the meta‐analyses, publication bias was inspected visually through a funnel plot and further quantified by Egger's test (Page et al. 2024). A fill‐and‐trim analysis was conducted when Egger's test suggested significant publication bias (*p* < 0.05). All statistical analyses were based on two‐sided statistical tests, and statistical significance was defined as a *p*‐value below 0.05.

## 3 Results

### 3.1 Search Outcome

**FIGURE 1:**
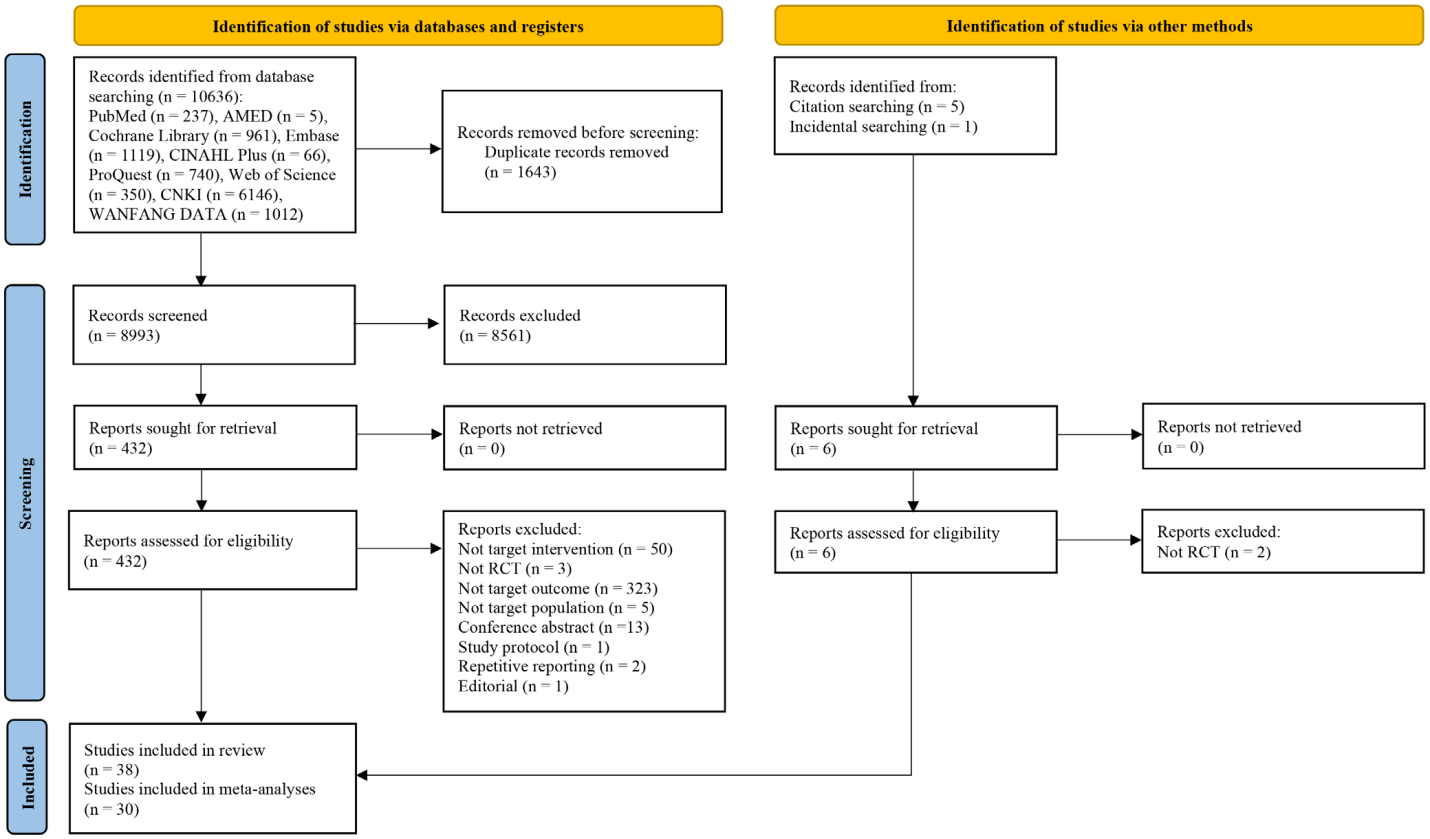
PRISMA flow chart. [Colour figure can be viewed at wileyonlinelibrary.com]

Following the elimination of 1643 duplicate entries and the exclusion of 8561 irrelevant records, this review included 38 eligible studies from a total of 438 records after a thorough examination of the full texts. The search process is illustrated in the PRISMA Flowchart (Figure 1).

### 3.2 Qualitative Synthesis

#### 3.2.1 Study Participants

We included 38 RCTs that encompassed 2995 LC patients. These studies were published predominantly in the last 5 years, with publication dates ranging from 2011 to 2024. Table 1 summarises the characteristics of the included studies. Thirty‐four trials were conducted in China, and four were implemented in the UK, Vietnam, Australia, and Germany, respectively. The average ages of the participants ranged from 50 to 69 years old (four studies did not report the mean age), with disease stages spanning from early to advanced. Ten trials enrolled participants experiencing cough at baseline.

**TABLE 1:** Study characteristics.

Author/Year/Country	Participants (EG/CG)	Sample size (EG/CG)	Intervention		Control	Key outcomes	Adverse events	Attrition (%)
			Content	Duration				
Wang et al./2024/China	Age 60 ± 10: 62 ± 10 Sex (F) 25 (47.2%): 30 (58.8%) NSCLC patients undergoing surgery	53/51	TEAS	5 days	Sham TEAS	Cough occurrence VAS (cough) LCQ	No	5.5
Cen/2022/China	Age 62.8 ± 8.9: 61.9 ± 9.1 Sex (F) 8 (14.3%): 19 (31.7%) LC patients undergoing radiotherapy	56/60	TEAS + Acupuncture	6 weeks	NT	TCM symptom score EORTC QLQ‐C30 (global health)	No	9.4
Zhang et al./2024/China	Age 58.9 ± 10.8: 58.5 ± 11.6 Sex (F) 30 (46.2%): 30 (46.2%) LC patients with cough	65/65	Acupuncture + UC	1 week	UC	MDASI‐LC (cough) MDASI‐LC (expectoration) MDASI‐LC (shortness of breath)	Tape allergy	0
Chen et al./2024/China	Age NR Sex (F) 13 (43.3%): 14 (46.7%) Postoperative LC patients with chronic cough	30/30	Acupuncture + WM	8 weeks	WM	LCQ Unspecified instrument (cough)	NR	0
Liu et al./2021/China	Age 50.2 ± 6.9: 50.8 ± 7.8 Sex (F) 22 (61.1%): 19 (52.8%) Postoperative LC patients with chronic pain	36/36	Acupuncture + WM	4 weeks	WM	TCM symptom score	NR	0
Ding & Chen/2023/China	Age 58.7 ± 5.1: 56.7 ± 5.1 Sex (F) 13 (43.3%): 14 (46.7%) Postoperative LC patients with chronic cough	30/30	Opening and Closing Six‐Qi acupuncture + WM	2 weeks	WM	CSS LCQ	NR	0
Zhao/2019/China	Age 50.3 ± 7.6: 50.6 ± 8.7 Sex (F) 15 (53.6%): 12 (42.9%) Postoperative LC patients with chronic pain	28/28	Plum blossom acupuncture + WM	4 weeks	WM	TCM symptom score	No	6.7
Zhu et al./2022/China	Age 56.3 ± 11.9: 58.8 ± 11.3 Sex (F) 9 (22.5%): 7 (17.5%) Postoperative NSCLC patients with chronic cough	14/20	Electroacupuncture	4 weeks	NT	EORTC QLQ‐C30 (global health) EORTC‐LC13 (dyspnea) LCQ	NR	15.0
Jiang/2022/China	Age 56.0 ± 10.5 (total sample) Sex (F) 42 (71.2%): 36 (61.0%) LC patients undergoing surgery	59/59	Auricular acupressure + UC	1 week	UC	Modified MDASI‐LC FACT‐L Incidence of symptoms	NR	3.3
Wu/2023/China	Age 30 to 73 (mean age not reported) Sex (F) 18 (42.8%): 20 (47.6%) LC patients	42/42	Acupoint embedding +WM	2 months	WM	Unspecified instrument (cough)	NR	0
Shi et al./2018/China	Age 54: 60 (means) Sex (F) 13 (43.3%): 15 (50%) LC patients with pleural effusion	30/30	Acupuncture + Acupoint application	9 weeks	Acupoint application	MRC‐DS Unspecified instrument (cough)	NR	0
Tian/2020/China	Age 60.5 ± 9.2: 62.1 ± 7.6 Sex (F) 7 (29.2%): 5 (21.7%) Advanced NSCLC patients receiving chemotherapy	24/23	Acupuncture + Moxibustion + CM	6 weeks	CM	EORTC‐QLQ C30 TCM symptom score	No	6
Zhou et al./2024/China	Age 56.9 ± 6.1: 56.8 ± 6.0 Sex (F) 8 (26.7%): 10 (33.3%) LC patients	30/30	Acupoint patting + Moxibustion + UC	10 days	UC	TCM symptom score	NR	0
Yin/2023/China	Age 64.7 ± 8.0: 63.0 ± 8.5 Sex (F) 12 (40.0%): 12 (40.0%) LC patients underwent no anti‐cancer treatments	30/30	Moxibustion + CM	8 weeks	CM	TCM symptom score	No	14.3
Zhang et al./2022/China	Age 60.5 ± 10.1: 62.6 ± 8.3 Sex (F) 8 (26.7%): 8 (26.7%) Patients with LC cough	30/30	Moxibustion + WM + CM	2 weeks	WM + CM	CSS VAS (cough) LCQ	Mild anaphylaxis	0
Xu/2016/China	Age 66.3 ± 6.9: 66.5 ± 6.0 Sex (F) 13 (43.3%): 13 (43.3%) Advanced NSCLC patients with cough and expectoration	30/30	Moxibustion + WM + CM	2 weeks	WM + CM	TCM symptom score	No	6.25
You/2019/China	Age 61.3 ± 7.7: 62.4 ± 7.1 Sex (F) 16 (48.5%): 15 (45.5%) NSCLC patients	33/33	Moxibustion + CM	3 months	CM	TCM symptom score	NR	0
Wang et al. (a)/2023/China	Age 59.2 ± 6.8: 58.7 ± 6.5 Sex (F) 12 (40.0%): 11 (36.7%) LC patients	30/30	Moxibustion + CM	8 weeks	CM	Self‐designed TCM symptom score	NR	0
Dong/2020/China	Age 57.4 ± 16.3: 57.3 ± 17.3 Sex (F) 9 (25.7%): 11 (32.4%) Advanced NSCLC patients receiving chemotherapy	35/34	Moxibustion + CM + UC	3 weeks	CM + UC	TCM symptom score	NR	2.8
Chen et al./2020/China	Age 53.6 ± 9.5: 51.32 ± 8.4 Sex (F) 21 (48.8%): 17 (39.5%) Advanced LC patients with cough	38/40	Moxibustion + UC	5–7 days	UC	VAS (cough) LCQ	NR	9.3
Gao/2018/China	Age NR Sex (F) 7 (35.0%): 9 (42.9%) LC patients with receiving chemotherapy	20/21	Moxibustion + UC	6 weeks	UC	TCM symptom score	NR	6.8
Lin et al./2024/China	Age 58.7 ± 8.8: 59.9 ± 9.4 Sex (F) 22 (50.0%): 19 (45.2%) Advanced LC patients with cough	44/42	Moxibustion + CM	2 weeks	CM	VAS (cough) CSS	NR	8.5
Guo et al./2024/China	Age 60.5 ± 1.2: 60.2 ± 2.6 Sex (F) 27 (46.6%): 28 (48.3%) LC patients undergoing radiotherapy	58/58	Pulmonary rehabilitation + UC	4 weeks	UC	MSAS EORTC QLQ‐C30	NR	0
He et al./2021/China	Age 64.4 ± 6.6: 65.2 ± 6.1 Sex (F) 14 (31.8%): 18 (41.9%) Postoperative NSCLC patients undergoing radiotherapy	44/43	Pulmonary rehabilitation + UC	24 weeks	UC	MSAS EORTC QLQ‐C30	NR	0
Lin et al./2023/China	Age 58.7 ± 3.5: 58.8 ± 3.4 Sex (F) 17 (42.5%): 19 (47.5%) LC patients undergoing radiotherapy	40/40	Pulmonary rehabilitation + UC	3 months	UC	MSAS EORTC QLQ‐C30	NR	0
Ma/2022/China	Age 69.0 ± 2.2: 69.4 ± 2.3 Sex (F) 9 (30%): 12 (40%) LC patients undergoing surgery	30/30	Pulmonary rehabilitation + UC	1 week	UC	Self‐designed TCM symptom score	NR	0
Zhao et al./2013/China	Age 58.8 ± 6.1: 58.4 ± 7.8 Sex (F) 14 (29.2%): 13 (26%) LC patients undergoing surgery	48/50	Pulmonary rehabilitation	1 week	NT	EORTC‐QLQ C30 EORTC‐QLQ LC13	NR	0
Zhou & Ji/2024/China	Age 56.9 ± 4.6: 55.6 ± 4.3 Sex (F) 20 (37.0%): 24 (44.4%) LC patients undergoing surgery	54/54	Self‐management approach + UC	12 weeks	UC	CSS FACT‐L	NR	0
Zheng & Wan/2020/China	Age 56.3 ± 11.7: 55.8 ± 10.1 Sex (F) 14 (37.8%): 13 (35.1%) LC patients receiving chemotherapy and presenting cough	37/37	Self‐management approach + UC	NR	UC	CDS CSS EORTC QLQ‐C30	NR	0
Wang et al. (b)/2023/China	Age 57.3 ± 8.6: 58.4 ± 8.0 Sex (F) 34 (68.0%): 29 (58.0%) Early‐stage NSCLC patients undergoing surgery and presenting fatigue, shortness of breath, cough, pain and anorexia	50/50	Self‐management approach	5 days	UC	CSS Borg Scale EORTC QLQ‐C30	NR	0
Shi et al./2024/China	Age 68.6 ± 3.4: 68.6 ± 3.5 Sex (F) 14 (32.6%): 16 (37.2%) LC patients receiving chemotherapy	43/43	Multi‐component (emotional support + moxibustion + message + ear acupressure + dietary support) + UC	4 weeks	UC	TCM symptom score	NR	0
Yorke et al./2015/UK	Age 67.8 ± 10.1: 67.6 ± 9.1 Sex (F) 28 (56.0%): 26 (51.0%) LC patients with dyspnea‐cough‐fatigue symptom cluster	31/40	Multi‐component (breathing exercise + acupressure + coughing ease + education) + UC	12 weeks	UC	Dysponea‐12 NRS (breathlessness) MCLC EQ‐5D‐3L	NR	29.7
Henke et al./2014/Germany	Age NR Sex (F) NR Advanced NSCLC patients	18/11	Physical exercise (aerobic + strengthening exercise) + UC	9–12 weeks	UC	EORTC‐QLQ C30 EORTC‐QLQ LC13	NR	37.0
Granger et al./2024/Austratia	Age 65.4 ± 10.8: 67.5 ± 8.1 Sex (F) 33 (56.9%): 35 (60.3%) Postoperative NSCLC patients	56/56	Physical exercise (aerobic and strengthening exercise supported by phone consultations) + UC	3 months	UC	EORTC‐QLQ C30 EORTC‐QLQ LC13	New calf pain	11.2
Molassiotis et al./2021/Vietnam	Age 57.6 ± 9.6: 56.1 ± 9.3 Sex (F) 19 (24.4%): 21 (26.9%) LC patients with dyspnea‐fatigue‐anxiety symptom cluster	78/78	Physical exercise (Qigong) + UC	6 weeks	UC	CDS MCLC EORTC QLQ‐C30	No	26.9
Su et al./2024/China	Age 53.6 ± 11.4: 56.2 ± 12.3 Sex (F) 35 (66.0%): 27 (50.9%) NSCLC patients undergoing surgery	51/50	Physical exercise (Qigong) + UC	12 weeks	UC	NRS (cough) NRS (dyspnea)	No	4.7
Li et al./2011/China	Age 57 ± 5.0 (total sample) Sex (F) 36.1 (total sample) Advanced NSCLC patients with pleural effusion	36/36	Psychoeducational support (psychological care + health education)	1 month	UC	Self‐designed quality of life questionnaire	NR	0
Qin et al./2019/China	Age 60.5 ± 7.6: 56.1 ± 10.1 Sex (F) 18 (56.3%): 22 (68.8%) Postoperative LC patients	32/32	Mindfulness intervention + CM	3 months	CM	FACT‐L TCM symptom score	No	0

Abbreviations: CDS, Cancer Dyspnea Scale; CG, control group; CM, Chinese medicine; CSS, cough symptom score; EG, experimental group; EORTC QLQ‐C30, European Organisation for Research and Treatment of Cancer Core Quality of Life Questionnaire; EORTC QLQ‐LC13, European Organisation for Research and Treatment of Cancer Quality of Life Questionnaire‐Lung Cancer 13; EQ‐5D‐3L, EuroQoL 5‐Dimension 3‐Level; FACT‐L, Functional Assessment of Cancer Therapy‐Lung; LC, lung cancer; LCQ, Leicester Cough Questionnaire; MCLC, Manchester Cough in Lung Cancer; MDASI‐C, MD Anderson Symptom Inventory‐Core; MDASI‐LC, MD Anderson Symptom Inventory‐Lung Cancer; MRC‐DS, Medical Research Council Dyspnea Scale; MSAS, Memorial Symptom Assessment Scale; NR, not reported; NRS, Number Rating Scale; NSCLC, non‐small cell lung cancer; NT, no treatment; TCM, Traditional Chinese Medicine; TEAS, transcutaneous Electrical Acupuncture Stimulation; UC, usual care; VAS, Visual Analogue Scale; WM, western medicine.

#### 3.2.2 Interventions and Control Conditions

The 38 NPIs included acupuncture therapy (*N* = 11) (Cen 2022; Chen et al. 2024; Ding and Chen 2023; Jiang 2022; Liu et al. 2021; Shi et al. 2018; Wang et al. 2024; Wu 2023; Zhang et al. 2024; Zhao 2019; Zhu et al. 2022), moxibustion (*N* = 9) (Chen et al. 2020; Dong 2020; Gao 2018; Lin et al. 2024; Wang 2023; Xu 2016; Yin et al. 2023; Zhang, Liu, et al. 2022), acupuncture therapy combined with moxibustion (*N* = 2) (Tian 2020; Zhou, Dai, et al. 2024), pulmonary rehabilitation (*N* = 5) (Guo et al. 2024; He et al. 2021; Lin et al. 2023; Ma 2022; Zhao and Zheng 2013), self‐management intervention (*N* = 3) (Wang et al. 2023; Zheng and Wan 2020; Zhou and Ji 2024), physical exercise (i.e., Qigong and conventional exercise modalities) (*N* = 4) (Granger et al. 2024; Henke et al. 2014; Molassiotis et al. 2021; Su et al. 2024), psychoeducational support (*N* = 1) (Li et al. 2011), mindfulness intervention (*N* = 1) (Qin 2019), and multicomponent interventions (*N* = 2) (Shi et al. 2024; Yorke et al. 2015). Of these 38 interventions, 16 combined NPIs with Western and/or Chinese medicine, while their control group was usual care involving Western and/or Chinese medicine alone. For the treatment durations, most studies (*N* = 21) implemented the interventions for longer than 30 days, but 16 studies adopted a treatment plan for no more than 30 days. One study did not clarify its treatment period (Zheng and Wan 2020). Of the 21 studies that documented the intervention providers, 13 implemented nurse‐led interventions, and six applied interventions provided by doctors, and two by physiotherapists. For the comparators, only one employed placebo control using sham transcutaneous electrical acupoint stimulation (TEAS) (Wang et al. 2024); the remaining studies used controls involving usual care or no treatment.

#### 3.2.3 Outcome Measurement

Cough was assessed through cough severity (*N* = 35), cough occurrence (*N* = 2), and cough‐related quality of life (*N* = 7) post‐intervention, 1 month or 3 months after treatment. None of the identified studies reported cough frequency. Cough severity was measured by the Visual Analog Scale/Numeric Rating Scale (VAS/NRS) (*N* = 5), Cough Symptom Score (CSS) (*N* = 6), Manchester Cough in Lung Cancer (MCLC) Scale (*N* = 2), Traditional Chinese Medicine (TCM) Symptom Score (*N* = 12), Modified MD Anderson Symptom Inventory‐Lung Cancer (MDASI‐LC) (*N* = 2), Memorial Symptom Assessment Scale (MSAS) (*N* = 3), European Organisation for Research and Treatment of Cancer Quality of Life Questionnaire‐Lung Cancer 13 (EORTC QLQ‐LC13) (*N* = 3), and self‐designed/unspecified instruments (*N* = 5). Cough occurrences were reported in two studies by calculating the number of patients experiencing cough divided by the number of patients included in the group. Studies that evaluated the cough‐related quality of life consistently used the Leicester Cough Questionnaire (LCQ) (*N* = 6).

For secondary outcomes, expectoration was evaluated by 15 RCTs using modified MDASI‐LC (*N* = 2), TCM Symptom Score (*N* = 10) or self‐designed instruments (*N* = 2), and 25 RCTs assessed dyspnea using the modified MDASI‐LC (*N* = 2), TCM Symptom Score (*N* = 8), Cancer Dyspnea Scale (CDS) (*N* = 3), European Organisation for Research and Treatment of Cancer Quality of Life Core Questionnaire (EORTC QLQ‐C30) (*N* = 2), EORTC QLQ‐LC13 (*N* = 4), Dyspnea‐12 (*N* = 1), Borg Scale (*N* = 1), Modified Medical Research Council Dyspnea Scale (MRC‐DS) (*N* = 1), or self‐designed/unspecified instruments (*N* = 3). Measures for general quality of life were mostly EORTC QLQ‐C30 (*N* = 13), followed by Functional Assessment of Cancer Therapy‐Lung (FACT‐L) (*N* = 3), EuroQoL 5‐Dimension 3‐Level (EQ‐5D‐3L) (*N* = 1), and self‐designed quality of life questionnaire (*N* = 1).

#### 3.2.4 Adverse Events

While most studies (*N* = 26) did not mention adverse events, nine studies (23.7%) found no adverse events and three studies (7.9%) reported minimal adverse events. These included tape allergy associated with acupuncture (Zhang et al. 2024), mild anaphylaxis observed in combined interventions that included moxibustion, Chinese medicine, and Western medicine (Zhang, Liu, et al. 2022), and new calf pain reported for aerobic and strengthening exercise (Granger et al. 2024).

#### 3.2.5 Retention

Concerning the retention rates, 21 studies (55.2%) reported no attrition, 11 studies (28.9%) had an attrition below 10% (Chen et al. 2020; Dong 2020; Jiang 2022; Wang et al. 2024; Xu 2016), and six studies (15.7%) observed an attrition of 11.2%–37.0% (Molassiotis et al. 2021; Yorke et al. 2015; Zhu et al. 2022; Zong 2020). The highest attrition (26.9%–37.0%) was reported in three RCTs that incorporated predominantly advanced LC patients, of which two adopted physical exercise (Henke et al. 2014; Molassiotis et al. 2021) and one employed a multicomponent intervention (Yorke et al. 2015).

#### 3.2.6 Quality Appraisal

**FIGURE 2:**
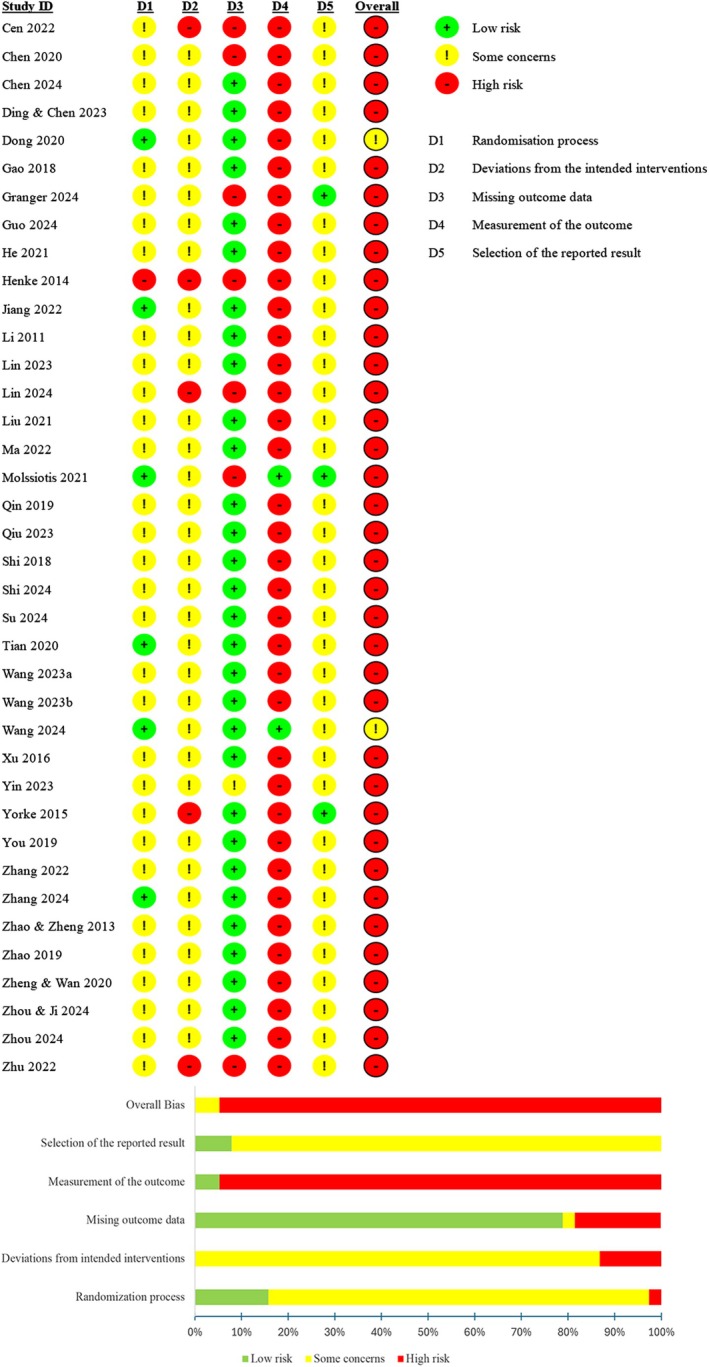
Summary of risk of bias. [Colour figure can be viewed at wileyonlinelibrary.com]

Figure 2 illustrates that 36 of the 38 included studies (94.7%) were rated high risk overall, mostly due to the high risk in outcome measurement. This is linked to the absence of blinding among participants who served as the assessors of the self‐reported outcomes (i.e., cough). Some concerns arise with selective reporting bias because most studies (*N* = 35) did not pre‐specify their research intentions. The risk of bias induced by deviations from the intended interventions was rated as high in five studies (13.2%) and some concerns in 33 studies (86.8%) due to the lack of blinding among the participants and intervention providers. Bias related to the randomisation process was low in six studies (15.8%), with most of the remaining (81.6%) having some concerns and one (2.6%) having high risk. A relatively low risk of bias was associated with missing data, as 30 studies (78.9%) were graded as low risk in this domain.

### 3.3 Meta‐Analysis Results

**FIGURE 3:**
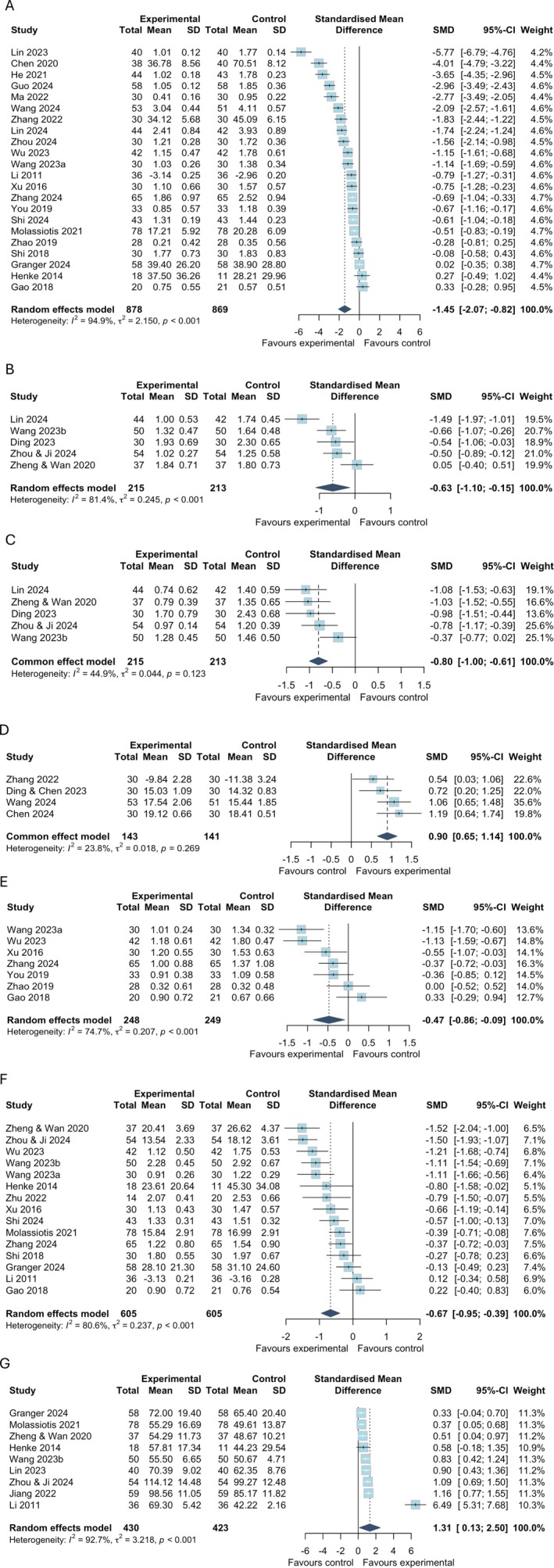
Forest plots. (A) cough severity (no CSS); (B) cough severity (CSS‐D); (C) cough severity (CSS‐N); (D) cough‐related quality of life; (E) expectoration; (F) dyspnea; (G) general quality of life. CI, confidence interval; CSS, cough symptom score; CSS‐D, cough symptom score‐daytime; CSS‐N, cough symptom score‐nighttime; SD, standard deviation; SMD, standardised mean difference. [Colour figure can be viewed at wileyonlinelibrary.com]

Among the 38 RCTs being reviewed, 30 RCTs were included in the meta‐analyses (27 on cough severity, 4 on cough‐related quality of life, 7 on expectoration, 15 on dyspnea, and 9 on general quality of life). Cough occurrence was assessed in only two studies employing acupuncture therapy, which displayed heterogeneous effects (*I* ^*2*^ = 64.9%) (Jiang 2022; Wang et al. 2024), precluding a meta‐analysis. Eight RCTs (Cen 2022; Dong 2020; Liu et al. 2021; Qin 2019; Su et al. 2024; Tian 2020; Yin et al. 2023; Yorke et al. 2015) with data descriptions unsuitable for pooling (presenting mean change or median values only) were excluded from the meta‐analysis along with one study that displayed disproportionate effects on all the outcomes investigated (i.e., cough severity, dyspnea severity, general quality of life) (Zhao and Zheng 2013). Two studies were removed from the pooling of cough‐related quality of life, one with missing data and the other presenting dubious data that were inconsistent with the text description (Chen et al. 2020). Table 2 and Figure 3 display the results of meta‐analyses, forest plots, sensitivity analyses, and publication bias.

**TABLE 2:** Summary of the meta‐analyses.

Outcome	*k*	*n* (EG/CG)	Effect size (SMD; 95% CI)	*p*	Heterogeneity		Publication bias (Egger's test *p*)	Adjusted effect size (SMD; 95% CI)
					*I* ^2^ (%)	*p*		
Cough severity (No CSS)	22	878/869	−1.45 [−2.07, −0.82]	< 0.001	94.9	< 0.001	0.003	−1.10 [−1.86, −0.34]
Cough severity (CSS‐D)	5	215/213	−0.63 [−1.10, −0.14]	0.01	81.4	< 0.001	—	—
Cough severity (CSS‐N)			−0.80 [−1.00, −0.61]	< 0.001	44.9	0.12	—	—
Cough‐related quality of life	4	143/141	0.90 [0.65, 1.14]	< 0.001	23.8	0.27	—	—
Expectoration	7	248/249	−0.47 [−0.86, −0.09]	0.02	74.7	< 0.001	—	—
Dyspnea	15	605/605	−0.67 [−0.95, −0.39]	< 0.001	80.6	< 0.001	0.46	—
General quality of life	9	430/423	1.31 [0.13, 2.50]	0.03	92.7	< 0.001	—	—

*Note:* Em dash indicates no analysis was conducted.

Abbreviations: CG, control group; CI, confidence interval; CSS, cough symptom score; CSS‐D, cough symptom score‐daytime; CSS‐N, cough symptom score‐nighttime; EG, experimental group; *k*, number of studies; *n*, number of participants; SMD, standardised mean difference.

#### 3.3.1 Effects on Cough Severity

Twenty‐seven studies were included for the meta‐analysis of cough severity (acupuncture therapy: *N* = 6; moxibustion: *N* = 9; acupuncture combined with moxibustion: *N* = 1; pulmonary rehabilitation: *N* = 4; self‐management approach: *N* = 2; multicomponent intervention: *N* = 1; psychoeducational support: *N* = 1; and physical exercise: *N* = 3), with 22 reporting an overall score and 5 providing daytime and nighttime cough scores.

The meta‐analysis of 22 RCTs reporting an overall cough severity score revealed a substantial mean effect size, albeit with considerable heterogeneity (SMD: −1.45; 95% CI: −2.07 to −0.82; *p* < 0.001; *I* ^*2*^ = 94.9%; 1747 participants) (Figure 3A). As Table 3 shows, the meta‐regression analysis suggested nonsignificant effects on heterogeneity for all pre‐specified potential covariates except intervention type (*p* < 0.001). The pre‐specified subgroup analysis further indicated that pulmonary rehabilitation demonstrated a greater positive effect (SMD: −3.75; 95% CI: −5.04 to −2.45; *I* ^*2*^ = 88.9%) than acupuncture and moxibustion therapies (SMD: −0.86; 95% CI: −1.66 to −0.16; *I* ^*2*^ = 90.3% and SMD: −1.39; 95% CI: −2.37 to −0.40; *I* ^*2*^ = 93.3%), while physical exercise interventions showed nonsignificant improvement (SMD: −0.15; 95% CI: −0.59 to 0.30; *I* ^*2*^ = 68.6%).

**TABLE 3:** Summary of meta‐regression and subgroup analyses.

Outcome	Meta‐regression		Subgroups	*k*	*n* (EG/CG)	Effect size (SMD; 95% CI)	Between‐group heterogeneity	
	Potential covariates	*p*					*I* ^2^ (%)	*p*
Cough severity (*N* = 22)	Country	0.07	—	—	—	—	—	—
	Intervention type	< 0.001	Acupuncture therapy	5	218/216	−0.86 [−1.56, −0.16]	90.3	< 0.001
			Moxibustion	7	225/226	−1.39 [−2.37, −0.40]	93.3	
			Physical exercise	3	154/147	−0.15 [−0.59, 0.30]	68.6	
			Pulmonary rehabilitation	4	172/171	−3.75 [−5.04, −2.45]	88.9	
	Instrument validity	0.52	—	—	—	—	—	—
	Population	0.34	—	—	—	—	—	—
	Comparator	0.52	—	—	—	—	—	—
	Treatment duration	0.47	—	—	—	—	—	—
Expectoration (*N* = 7)	Country	—	—	—	—	—	—	—
	Intervention type	—	Acupuncture therapy	3	135/135	−0.50 [−1.14, 0.13]	82.0	0.89
			Moxibustion	4	113/114	−0.45 [−1.02, 0.13]	76.2	
	Population	—	Coughing participants	2	95/95	−0.43 [−0.72, −0.14]	0.0	0.89
			Participants not necessarily presenting baseline cough	5	153/154	−0.48 [−1.05, 0.10]	82.7	
	Comparator	—	Contained WM/CM	5	163/163	−0.64 [−1.08, −0.21]	72.8	0.17
			Not contained WM/CM	2	85/86	−0.07 [−0.75, 0.61]	73.4	
	Treatment duration	—	≤ 30 days	3	123/123	−0.33 [−0.58, −0.08]	12.6	0.47
			> 30 days	4	125/126	−0.59 [−1.27, 0.08]	83.6	
Dyspnea (*N* = 15)	Country	0.34	—	—	—	—	—	—
	Intervention type	0.01	Acupuncture therapy	4	151/157	−0.65 [−1.09, −0.20]	70.2	< 0.001
			Moxibustion	3	80/81	−0.53 [−1.28, 0.22]	80.4	
			Physical exercise	3	154/147	−0.32 [−0.55, −0.10]	25.6	
			Self‐management intervention	3	141/141	−1.36 [−1.62, −1.10]	3.3	
	Instrument validity	0.27	—	—	—	—	—	—
	Population	0.53	—	—	—	—	—	—
	Comparator	0.54	—	—	—	—	—	—
	Treatment duration	0.74	—	—	—	—	—	—
General quality of life (*N* = 9)	Country	—	China	5	239/239	2.05 [−0.04, 4.13]	95.1	0.12
			Other countries	3	154/147	0.37 [0.14, 0.61]	0.0	
	Intervention type	—	Physical exercise	3	154/147	0.37 [0.14, 0.60]	0.0	0.02
			Self‐management intervention	3	141/141	0.84 [0.59, 1.08]	42.4	
	Instrument validity	—	—	—	—	—	—	—
	Population	—	—	—	—	—	—	—
	Comparator	—	—	—	—	—	—	—
	Treatment duration	—	≤ 30 days	3	145/145	2.79 [−0.77, 6.35]	97.5	0.24
			> 30 days	5	248/241	0.64 [0.32, 0.97]	64.8	

*Note:* Em dash indicates no analysis was conducted.

Abbreviations: CG, control group; CI, confidence interval; CM, Chinese medicine; EG, experimental group; *k*, number of studies; *n*, number of participants; SMD, standardised mean difference; WM, Western medicine.

For the remaining five studies reporting daytime and nighttime cough scores (acupuncture therapy: *N* = 1; moxibustion: *N* = 1; self‐management approach: *N* = 3; 428 participants), the pooled results consistently indicated statistically significant improvements in daytime cough (SMD: −0.63; 95% CI: −1.10 to −0.15; *p* = 0.01; *I* ^*2*^ = 81.4%) and nighttime cough (SMD: −0.80; 95% CI: −1.00 to −0.61; *p* < 0.001; *I* ^*2*^ = 44.9%) (Figure 3B,C). Subgroup analysis was applied to only two pre‐specified potential covariates (i.e., population and comparator), with results suggesting that daytime cough was significantly improved in studies with LC patients not necessarily presenting baseline cough (SMD: −0.58; 95% CI: −0.86 to −0.30; *I* ^*2*^ = 0.0%, 208 participants) and the comparators involving Western/Chinese medicine (SMD: −1.02; 95% CI: −1.95 to −0.10; *I* ^*2*^ = 85.5%, 146 participants), but not in those in which LC patients presented cough at baseline, or the comparators did not include Western or/and Chinese medicine.

#### 3.3.2 Effects on Cough‐Related Quality of Life

Four studies were pooled for the meta‐analysis of cough‐related quality of life (acupuncture therapy: *N* = 3; moxibustion: *N* = 1). The pooled SMD indicated a significant improvement in cough‐related quality of life (SMD: 0.90; 95% CI: 0.65 to 1.14; *p* < 0.001; *I* ^*2*^ = 23.8%; 284 participants) (Figure 3D).

#### 3.3.3 Effects on Expectoration

Seven studies were included in the meta‐analysis of expectoration (acupuncture therapy: *N* = 3; moxibustion: *N* = 4). The pooled SMD suggested a small effect size in improving expectoration in LC (SMD: −0.47; 95% CI: −0.86 to −0.09; *p* < 0.001; *I* ^*2*^ = 74.7%; 497 participants) (Figure 3E). The subgroup analyses shown in Table 3 suggested significantly beneficial effects in participants experiencing cough at baseline (SMD: −0.43; 95% CI: −0.72 to −0.14; *I* ^*2*^ = 0.0%; 190 participants), comparators involving Western or/and Chinese medicine (SMD: −0.64; 95% CI: −1.08 to −0.21; *I* ^*2*^ = 72.8%; 326 participants), and those with a treatment duration of 30 days or less (SMD: −0.33; 95% CI: −0.58 to −0.08; *I* ^*2*^ = 12.6%; 246 participants). In contrast, we found nonsignificant improvements in the subgroups including acupuncture therapy, moxibustion, participants not necessarily presenting cough, comparators not containing Western or/and Chinese medicine, and those with treatment durations of over 30 days.

#### 3.3.4 Effects on Dyspnea

Fifteen studies were included in the meta‐analysis of dyspnea (acupuncture therapy: *N* = 4; moxibustion: *N* = 3; self‐management approach: *N* = 3; physical exercise: *N* = 3; psychoeducational support: *N* = 1; and multicomponent intervention: *N* = 1). The pooled SMD demonstrated an overall effect favouring NPIs over the controls in managing dyspnea in LC patients (SMD: −0.67; 95% CI: −0.95 to −0.39; *p* < 0.001; *I* ^*2*^ = 80.6%; 1210 participants) (Figure 3F). The meta‐regression (Table 3) identified no significant impacts on overall effects for the pre‐specified potential covariates except for intervention type (*p* = 0.01). Significant improvements were observed for acupuncture therapy, physical exercise, and self‐management interventions (SMD: −0.32 to −1.36), but not for moxibustion.

#### 3.3.5 Effects on General Quality of Life

Nine RCTs were pooled for the meta‐analysis of general quality of life (acupuncture therapy: *N* = 1; self‐management approach: *N* = 3; pulmonary rehabilitation: *N* = 1; psychoeducational support: *N* = 1; and physical exercise: *N* = 3). The meta‐analyses were based on the EORTC QLQ‐C30 global health status score and the total score of FACT‐L and self‐designed measures. Pooled data revealed a significantly positive effect of NPIs on general quality of life (SMD: 1.31; 95% CI: 0.13 to 2.50; *p* = 0.03; *I* ^*2*^ = 92.7%; 853 participants) (Figure 3G). Subgroup analyses (Table 3) revealed significantly greater effects with self‐management intervention (SMD: 0.84; 95% CI: 0.59 to 1.08; *I* ^*2*^ = 42.4%) than physical exercise (SMD: 0.37; 95% CI: 0.14 to 0.60; *I* ^*2*^ = 0.0%). The effect estimate did not differ significantly for the country (*p* = 0.12) and treatment durations (*p* = 0.24); however, it is noted that studies with treatment durations longer than 30 days displayed significantly positive effects (SMD: 0.64; 95% CI: 0.32 to 0.97; *I* ^*2*^ = 64.8%), while those that implemented a treatment duration ≤ 30 days showed nonsignificant improvements.

#### 3.3.6 Sensitivity Analyses, Publication Bias, and Certainty of Evidence

In the sensitivity analyses (See Figure S1), the effects regarding cough severity, cough‐related quality of life, and dyspnea remained significant after sequentially omitting each study. The sequential removal of three studies (Wang 2023; Wu 2023; Xu 2016) altered the effect estimates to nonsignificant for expectoration outcome, but no marked improvement in the overall heterogeneity was observed (*I* ^2^: 66.8%–78.9%). For the general quality of life outcome, sequentially omitting two studies led to nonsignificant effect estimates (Jiang 2022; Zhou and Ji 2024), but no improvement in the overall heterogeneity. However, it was noted that the study by Li et al. (2011) contributed to the heterogeneity; the exclusion of this study resulted in a considerable reduction in the overall heterogeneity (from 92.7% to 63.1%) and the effect estimate (SMD: 0.72; 95% CI: 0.48 to 0.96; *I* ^*2*^ = 63.1%).

The funnel plots (Figure S2) and Egger's regression analysis (Table 2) were applicable only to outcomes related to cough severity and dyspnea. The results indicated non‐significant publication bias for dyspnea (*p* = 0.46), but significant publication bias for cough severity (*p* = 0.003). A trim and fill analysis was performed for cough severity, and after adding two potential missing studies, the adjusted effect size decreased but remained statistically significant (SMD: −1.10; 95% CI: −1.86 to −0.34).

According to the GRADE guideline, the overall quality of evidence for NPIs in LC patients was rated as very low for cough severity, cough‐related quality of life, expectoration, dyspnea, and general quality of life (Table S2), primarily due to a high risk of bias in the included studies, significant heterogeneity across findings, variations in trial interventions, and imprecision related to limited sample size.

## 4 Discussion

This review is the first meta‐analysis to synthesise the evidence regarding the effect of NPIs on LC cough. The meta‐analyses indicated a favourable effect of NPIs in reducing cough severity, improving cough‐related quality of life, reducing cough‐related symptoms including expectoration and dyspnea, and improving overall health‐related quality of life. Meta‐regressions and subgroup analyses further revealed that intervention types significantly affected the efficacy of NPIs on cough severity, with pulmonary rehabilitation demonstrating the greatest effect size.

Several types of NPIs were identified from the included studies, including acupuncture therapy, moxibustion, pulmonary rehabilitation, self‐management interventions, physical exercise, psychoeducational support, mindfulness and multicomponent intervention. By quantitatively synthesising these approaches, this review provides an overview of NPI effectiveness for managing lung cancer cough. The meta‐analyses showed a small to large effect size in decreasing cough severity, with intervention type significantly influencing the effect size. Notably, pulmonary rehabilitation showed a greater effect than other NPIs (SMD: −3.75 vs. −0.20 to −1.33), whereas acupuncture and moxibustion therapies also demonstrated considerable benefits (SMD: 0.86 and 1.39). These findings provide critical insights for prioritising interventions and refining clinical guidelines. Cough in LC is thought to be due to excessive stimulation, increased sensitivity, or injuries to the neuronal pathways that occur within the airways or the central nervous system (Harle et al. 2012). Pulmonary rehabilitation may reduce cough through multifaceted strategies such as education, breathing and physical exercise, coughing techniques, and physiotherapy. These strategies in combination may address different factors contributing to cough, collectively reducing cough through multiple mechanisms, and ultimately leading to enhanced overall benefits (Luo et al. 2024). For instance, education and breathing exercise may increase central control of cough by modulating central inhibitory systems and may decrease peripheral sensitisation by avoiding cough triggers (Chamberlain Mitchell et al. 2019). The similar effect size of acupuncture and moxibustion therapies observed in this study could be ascribed to a parallel mechanism of action through acupoint stimulation (J. G. Lin et al. 2014). Their effects have been linked to cough sensitivity modulation via peripheral mechanisms, such as regulation of Transient Receptor Potential Vanilloid‐1 (TRPV‐1) pathway and anti‐inflammatory pathways (Xu et al. 2023; Zhu et al. 2022).

Four RCTs in our review that explored physical exercise reported inconsistent results. Notably, the two studies using Qigong (a mind–body exercise) both observed significant improvement in cough severity and general quality of life (Molassiotis et al. 2021; Su et al. 2024), whilst the two studies investigating conventional exercise (i.e., aerobic and strengthening exercise) consistently reported nonsignificant improvements in both outcomes. Similarly, a meta‐analysis of three quasi‐experimental trials of conventional exercise yielded a nonsignificant impact on cough in LC patients (Yang et al. 2020). The effect difference in cough between Qigong and traditional physical exercise is worth exploring. Mind–body exercise could produce a superior effect because of the combined effect of the multi‐components included (i.e., mindfulness, breathing exercises, and body movements), all of which may help to address cough through both central and peripheral mechanisms by strengthening respiratory muscles, reducing inflammation, enhancing immune function, and regulating stress and emotions (Feng et al. 2020). Nevertheless, given the limited evidence and inconsistent findings from the existing literature, further research is warranted to differentiate the effects of physical exercises of different modalities on LC cough. Additionally, relatively high attritions (26.9% and 37.0%) were reported in two studies investigating Qigong and conventional exercise that enrolled primarily advanced LC patients, while previous studies found high rates of completion (78% ‐ 85%) in tai‐chi and aeorbic exercise interventions among advanced LC patients with sleep disturbance (Cheung et al. 2021; Takemura et al. 2024). Future research may consider symptom‐based tailoring to optimize the adherence of advanced‐stage cancer patients when designing exercise interventions.

Three RCTs in this review adopting self‐management interventions (Wang et al. 2023; Zheng and Wan 2020; Zhou and Ji 2024) consistently reported reductions in cough severity, along with improvements in dyspnea. The self‐management interventions included in this review were all underpinned by knowledge‐attitude‐belief‐practice theory, delivered by nurses, and included targeted cough suppression elements such as cough education and breathing exercises. These components aim to improve patients' symptom management knowledge, coping skills, and behaviours and provide a practical framework for oncology nurses to integrate structured, nurse‐led self‐management strategies into care plans for patients with cough and dyspnea. Self‐management is a potential long‐term strategy for managing respiratory conditions (Taylor and Pinnock 2017). In recent years, there has been a growing focus on self‐management interventions in chronic respiratory conditions due to their positive impacts on health outcomes and their cost‐effectiveness (Ferreira et al. 2024; Rowntree and Hosseinzadeh 2022). Nevertheless, to conclude the effectiveness of self‐management interventions on LC cough and dyspnea, a larger body of evidence is required.

NPIs also showed a large effect size for improving cough‐related quality of life, consistent with a recent systematic review concluding that NPIs were associated with improvements in cough‐related quality of life in adults with chronic cough (Ilicic et al. 2022). This is unsurprising, considering the established positive correlation between cough severity and cough‐related quality of life (M. Zhang, Sykes, et al. 2022). In addition, NPIs showed significant benefits in managing cough‐related symptoms (i.e., expectoration and dyspnea) and in improving general quality of life in LC patients. While the results for dyspnea and general quality of life are consistent with previous literature (Gravier et al. 2022; Liu et al. 2019; Nguyen et al. 2023; Peddle‐McIntyre et al. 2019; Xi et al. 2022), our findings for expectoration are new addition to the literature. No review has focused on expectoration in LC patients, which may be attributed to the relatively lower priority placed on this particular outcome among LC patients, as they are generally faced with more prominent symptoms including pain, dyspnea, cough, fatigue, and sleep disturbance, as well as emotional distress (Karlsson et al. 2023). In fact, effective expectoration management is valuable in LC patients because it may benefit in preventing adverse events like pulmonary atelectasis and pulmonary infection (X.‐J. Guo et al. 2018). The collective benefits of NPIs in improving cough, expectoration, and dyspnea may also be explained through symptom cluster science. Earlier research consistently reported that respiratory symptoms including cough, dyspnea, and expectoration are closely linked to each other, often forming a symptom cluster in LC patients (Henoch et al. 2008; Ju et al. 2023; Ma et al. 2022). Improvement in any of the individual symptoms in a symptom cluster may improve the other symptom(s) (Kwekkeboom 2016). Future research could explore respiratory symptom clusters and the underlying mechanisms about how NPIs may work to improve these clusters in more depth.

The lack of objective cough measurement in the included studies is worthy of attention and represents an important methodological gap. Cough frequency, regarded as the gold standard objective measure of cough (Morice et al. 2020), was not reported by any of the studies we identified. Although cough monitor systems such as Leicester Cough Monitor and VitaloJAK have been recommended in the literature (Jakusova and Brozmanova 2023) and are commonly used in descriptive studies and antitussive clinical trials (Birring et al. 2017; Fukuhara et al. 2020; Holt and Smith 2023; Morice et al. 2021; Singh et al. 2022; Vertigan et al. 2016), our review shows that they are less frequently applied in research on non‐pharmacological cough management. Two studies reported cough occurrence, another outcome that can be measured objectively, but neither specified the tools used, leaving the objectivity of the data unknown. Future studies should comprehensively assess cough by adopting both subjective and objective measures (Jakusova and Brozmanova 2023; Wang et al. 2019).

Certain limitations exist with this review. First, despite efforts to minimise reporting bias through a thorough literature search, evidence of publication bias was detected, and substantial statistical heterogeneity persisted for cough severity, even after exploration via pre‐specified meta‐regression and subgroup analyses. These contributed to downgrading the certainty of evidence, limiting interpretation of the findings. Second, the literature search focused on primary outcomes, potentially introducing selection bias into the results of the secondary outcomes. Third, the review exclusively incorporated articles written in either Chinese or English, potentially introducing a language bias. Fourth, given that the studies predominantly focused on Chinese populations, the generalisability of the findings to other populations requires further investigation. Fifth, although this study found greater effects of pulmonary rehabilitation than other NPIs in improving cough, its superiority should be further determined by rigorously designed RCTs or a network meta‐analysis.

This review provides the first meta‐analytic evidence on the use of NPIs for managing cough in lung cancer, an area where clinical guidance has historically been limited. By demonstrating that NPIs are associated with minimal adverse events and offer potential benefits—not only for cough but also for expectoration, dyspnea, and overall quality of life—in patients with lung cancer, this review supports the integration of NPIs into clinical practice for cough and its associated symptoms. Specifically, clinical practitioners may consider employing pulmonary rehabilitation, typically encompassing psychoeducation, cough education, breathing exercise, and physical exercise, to improve cough and the related symptom cluster. Acupuncture and moxibustion therapies, although eliciting a smaller effect than pulmonary rehabilitation, emerge as viable options that can be selected based on resource availability and patient preferences. Additionally, the effects of NPIs on cough severity did not display substantial difference regardless of the presence of cough at baseline and integration of Western/Chinese medicine, indicating that NPIs may serve as both prophylactic and therapeutic treatments and could be implemented in combination with conventional medical treatments. These findings strengthen the evidence base for integrative symptom management in lung cancer care.

This review delineates key methodological and conceptual priorities to guide future studies in this field. In particular, by emphasising the predominant use of self‐reported outcomes in existing research, it underscores the need for more comprehensive cough assessment that combines subjective measures with objective indicators such as cough occurrence and frequency. Moreover, more rigorously designed research for each intervention type—particularly physical exercise and self‐management intervention—is required to strengthen the evidence base for their effectiveness and to inform clinical decision‐making. Furthermore, direct comparisons of different cough management and network meta‐analyses are needed to add knowledge concerning the superiority of intervention types. Additionally, more research can be conducted to explore the use of acupressure, a noninvasive self‐administered alternative to acpuncture, in cough management. This is because research has shown that acupressure may be potentially more effective than acupuncture in cancer symptom management (Cheung et al. 2022; Al Eleiwah et al. 2026) due to high accesibility and patient acceptance (Cheung et al. 2020, 2025). Finally, the study revealed the potential of NPIs to manage cough‐related symptom clusters, not just individual symptoms. This finding underscores the need for future research to explore innovative strategies that address cough within the broader context of symptom clusters. The observed effectiveness of interventions such as acupuncture, physical exercise, and self‐management interventions for both cough and dyspnea suggests promising avenues for targeted management of the cough‐dyspnea symptom cluster.

## 5 ;Conclusion

In conclusion, NPIs demonstrated significant overall benefits on cough (severity and cough‐related quality of life), expectoration, dyspnea, and general quality of life among LC patients, while the evidence on cough occurrence remains inconclusive. Pulmonary rehabilitation has shown the most promising effect. However, these results should be interpreted with caution due to low certainty of evidence. Further studies should consider adopting both subjective and objective cough measures. Additional research is required to ascertain the efficacy of some forms of NPIs, particularly physical exercise and self‐management strategies.

## Author Contributions

**Mengyao Cao:** conceptualisation, data curation, formal analysis, methodology, validation, writing – original draft, writing – review and editing. **Xueyan Cheng:** data curation, validation, writing – review and editing. **Victor Ho‐fun Lee:** validation, writing – review and editing. **Chia‐Chin Lin:** validation, writing – review and editing. **Denise Shuk Ting Cheung:** conceptualisation, methodology, validation, writing – review and editing.

## Funding

The authors have nothing to report.

## Disclosure

The authors have checked to make sure that our submission conforms as applicable to the Journal's statistical guidelines described here. The authors affirm that the methods used in the data analyses are suitably applied to their data within their study design and context, and the statistical findings have been implemented and interpreted correctly. The authors agree to take responsibility for ensuring that the choice of statistical approach is appropriate and is conducted and interpreted correctly as a condition to submit to the Journal.

## Conflicts of Interest

The authors declare no conflicts of interest.

## Supporting information


**Table S1:** Search strategy.
**Table S2:** GRADE assessment.
**Figure S1:** Sensitivity analyses. (A) cough severity (no CSS); (B) cough severity (CSS‐D); (C) cough severity (CSS‐N); (D) cough‐related quality of life; (E) expectoration; (F) dyspnea; (G) general quality of life. CI, confidence interval; CSS, cough symptom score; CSS‐D, cough symptom score‐daytime; CSS‐N, cough symptom score‐nighttime; SD, standard deviation; SMD, standardised mean difference.
**Figure S2:** Funnel plots. (A) cough severity; (B) cough severity (after trim and fill analysis); (C) dyspnea.

**Data S1:** jocn70289‐sup‐0002‐DataS1.docx.

## Data Availability

The data that supports the findings of this study are available in the Supporting Information of this article.
