# Supplementary material for: Non‐Pharmacological Interventions for Cough in Patients With Lung Cancer: A Systematic Review and Meta‐Analysis
Source: J Clin Nurs. 2026 Mar 15;35(8):3335–56. doi: 10.1111/jocn.70289 (PMC13353695; doi:10.1111/jocn.70289)

Table S1 Search strategy

| **Source** | **Search Strategy** |
| --- | --- |
| PubMed | ((lung neoplasm[MeSH Terms]) OR (lung cancer[Title/Abstract] OR lung neoplasm*[Title/Abstract] OR lung tumo*r[Title/Abstract] OR lung carcinoma[Title/Abstract] OR lung adenocarcinoma[Title/Abstract] OR lung malignan*[Title/Abstract])) AND ((cough[MeSH Terms]) OR (cough*[All Fields])) AND (random*[Title/Abstract]) |
| CINHAL-Plus  (via EBSCOhost) | S1 (MM "Lung Neoplasms+") OR (MM "Adenocarcinoma of Lung") OR (MM "Carcinoma, Non-Small-Cell Lung") Expanders - Apply equivalent subjects Search modes - Proximity  S2 (MM "Cough+") OR "cough" OR (MM "Chronic Cough") OR (MM "Whooping Cough") Expanders - Apply equivalent subjects Search modes - Proximity  S3 AB (lung cancer or lung neoplasms or lung tumo#r or lung adenocarcinoma or lung carcinoma or lung malignan*) Expanders - Apply equivalent subjects Search modes - Proximity  S4 TX cough* Expanders - Apply equivalent subjects Search modes - Proximity  S5 AB random* Expanders - Apply equivalent subjects Search modes - Proximity  S6 S1 OR S3 Expanders - Apply equivalent subjects Search modes - Proximity  S7 S2 OR S4 Expanders - Apply equivalent subjects Search modes - Proximity  S8 S5 AND S6 AND S7 Expanders - Apply equivalent subjects Search modes - Proximity |
| Cochrane | #1 MeSH descriptor: [Lung Neoplasms] explode all trees  #2 (lung neoplasm* OR lung tumo?r OR lung cancer OR lung malignan* OR lung carcinoma OR lung adenocarcinoma):ti,ab,kw  #3 MeSH descriptor: [Cough] explode all trees  #4 (cough*) (Word variations have been searched)  #5 (random*):ti,ab,kw (Word variations have been searched)  #6 (#1 OR #2) AND (#3 OR #4) AND #5 |
| EMBASE | Sources Embase, MEDLINE, Preprints  Query  ('lung cancer'/exp OR 'lung cancer' OR 'lung neoplasm':ab,ti,kw OR 'lung cancer':ab,ti,kw OR 'lung malignan*':ab,ti,kw OR 'lung carcinoma':ab,ti,kw OR 'lung tumo$r':ab,ti,kw OR 'lung adenocarcinoma':ab,ti,kw) AND ('coughing'/exp OR 'coughing' OR cough*) AND random*:ab,ti,kw |
| Web of Science | ((TS=(lung cancer OR lung carcinoma OR lung adenocarcinoma OR lung neoplasm OR lung tumor OR lung malignan*)) AND ALL=(cough*)) AND TS=(random*) |
| AMED  (via Ovid) | 1​ (lung neoplasm or lung cancer or lung tumo?r or lung carcinoma or lung adenocarcinoma or lung malignan*).mp. [mp=abstract, heading words, title]  2 exp Lung neoplasms/ or lung cancer.mp.  3 ​exp Cough/ or cough*.mp.  4 cough*.af.  5 random*.mp.  6 1 or 2  7 3 or 4  8 5 and 6 and 7 |
| ProQuest | abstract(lung cancer OR lung neoplasm* OR lung carcinoma OR lung adenocarcinoma OR lung malignan* OR lung tumor) AND fulltext(cough*) AND abstract(random*) |
| CNKI | TKA = ('肺癌' + '肺肿瘤' + '肺部肿瘤' + '肺部恶性肿瘤' + '肺恶性肿瘤' + '肺腺癌' + '肺鳞癌') AND FT = ('咳嗽' + '干咳') AND TKA = '随机' |
| Wangfang data | 检索表达式（中英文扩展&主题词扩展）：摘要:(肺癌 OR 肺部肿瘤 OR 肺肿瘤 OR 肺部恶性肿瘤 OR 肺恶性肿瘤 OR 肺腺癌 OR 肺鳞癌) and全部:(咳嗽 OR 干咳) and 摘要:(随机) |

Table S2 GRADE Assessment

| **Outcome** | **No. of studies (participants)** | **Risk of bias** | **Inconsistency** | **Indirectness** | **Imprecision** | **Publication bias** | **Hedges’ *g* (95% CI)** | **Certainty** |
| --- | --- | --- | --- | --- | --- | --- | --- | --- |
| Cough severity | 22 RCTs (1747) | ^a^Serious | ^b^Very Serious | ^c^Serious | Not Serious | Suspected | -1.45 [-2.07, -0.82] | ⊕⊝⊝⊝ **very low** |
| Cough severity (daytime) | 5 RCTs (428) | ^a^Serious | ^b^Serious | ^c^Serious | ^d^Serious | None | -0.63 [-1.10, -0.14] | ⊕⊝⊝⊝ **very low** |
| Cough severity (nighttime) | 5 RCTs (428) | ^a^Serious | Not Serious | ^c^Serious | ^d^Serious | None | -0.80 [-1.00, -0.61] | ⊕⊝⊝⊝ **very low** |
| Cough-related quality of life | 4 RCTs (284) | ^a^Serious | Not Serious | ^c^Serious | ^d^Serious | None | 0.90 [0.65, 1.14] | ⊕⊝⊝⊝ **very low** |
| Expectoration | 7 RCTs (497) | ^a^Serious | ^b^Serious | ^c^Serious | ^d^Serious | None | -0.47 [-0.86, -0.09] | ⊕⊝⊝⊝ **very low** |
| Dyspnea | 15 RCTs (1210) | ^a^Serious | ^b^Serious | ^c^Serious | Not serious | None | -0.67 [-0.95, -0.39] | ⊕⊝⊝⊝ **very low** |
| General quality of life | 9 RCTs (853) | ^a^Serious | ^b^Very Serious | ^c^Serious | Not serious | None | 1.31 [0.13, 2.50] | ⊕⊝⊝⊝ **very low** |

Note: RCTs, randomized controlled trials; CI, confidence interval;

^a^Most studies were rated as high risk of bias in methodology;

^b^Inconsistency: Substantial or considerable heterogeneity;

^c^Indirectness: Non-pharmacological interventions and the controls may vary in contents, mode of delivery and duration;

^d^Imprecision: Not met optimal information size.

Figure S1. Sensitivity analyses. A) cough severity (no CSS); B) cough severity (CSS-D); C) cough severity (CSS-N); D) cough-related quality of life; E) expectoration; F) dyspnea; G) general quality of life. SD, standard deviation; SMD, standardized mean difference; CI, confidence interval; CSS, cough symptom score; CSS-D, cough symptom score-daytime; CSS- N, cough symptom score-nighttime.

A


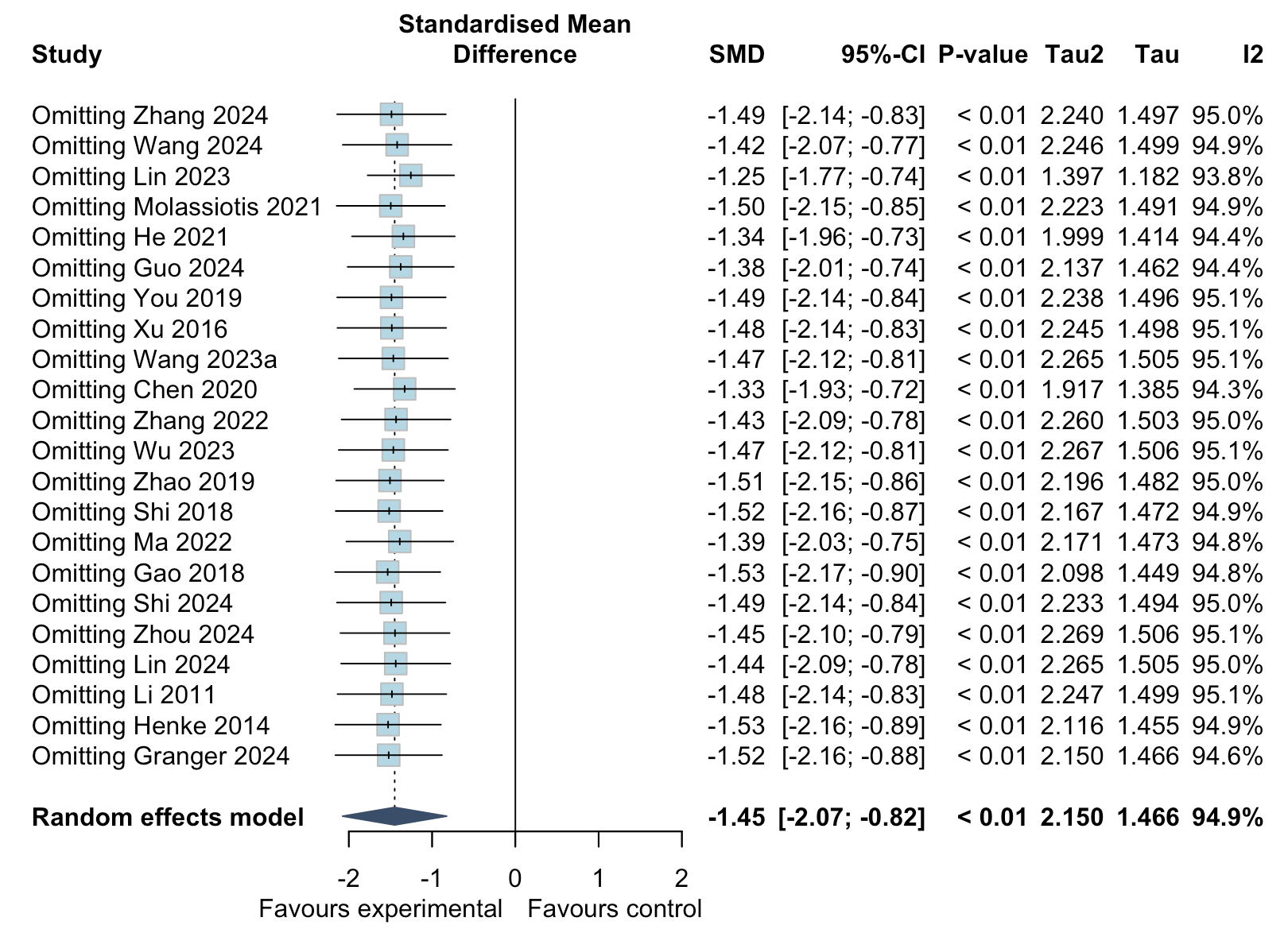


B


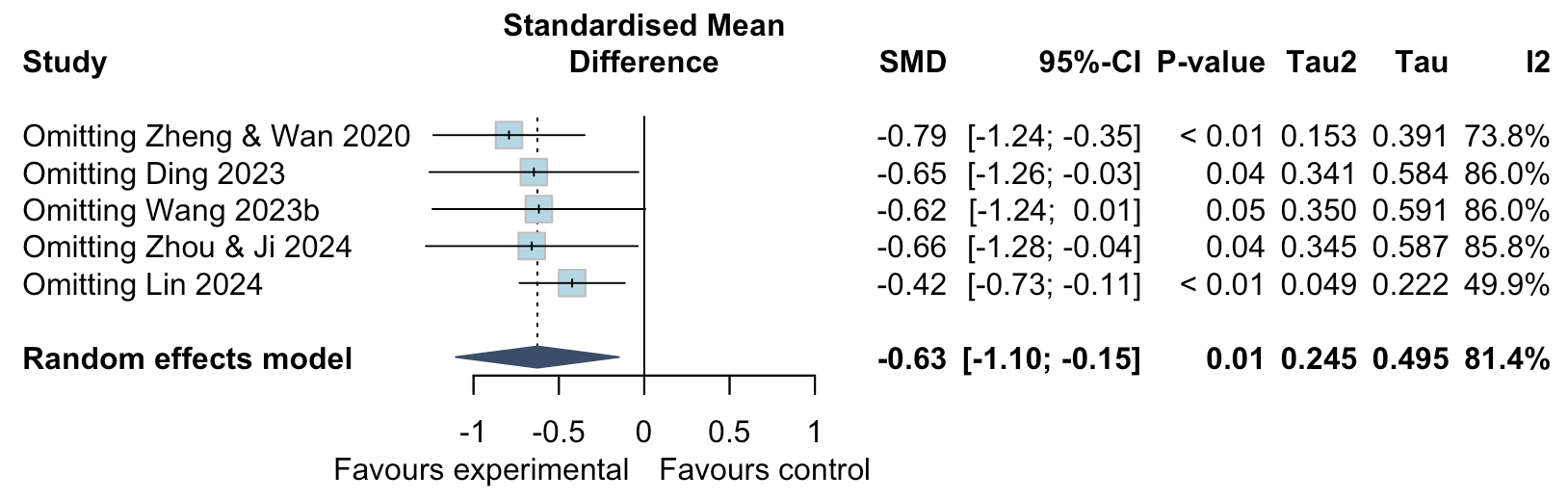


C


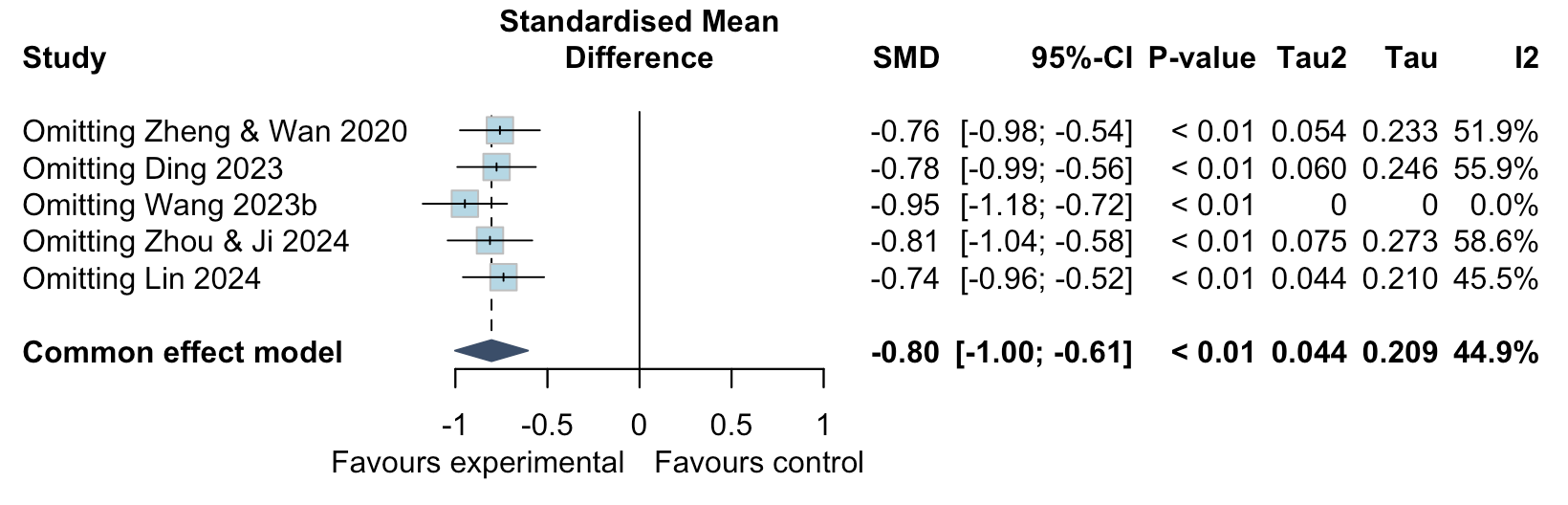


D


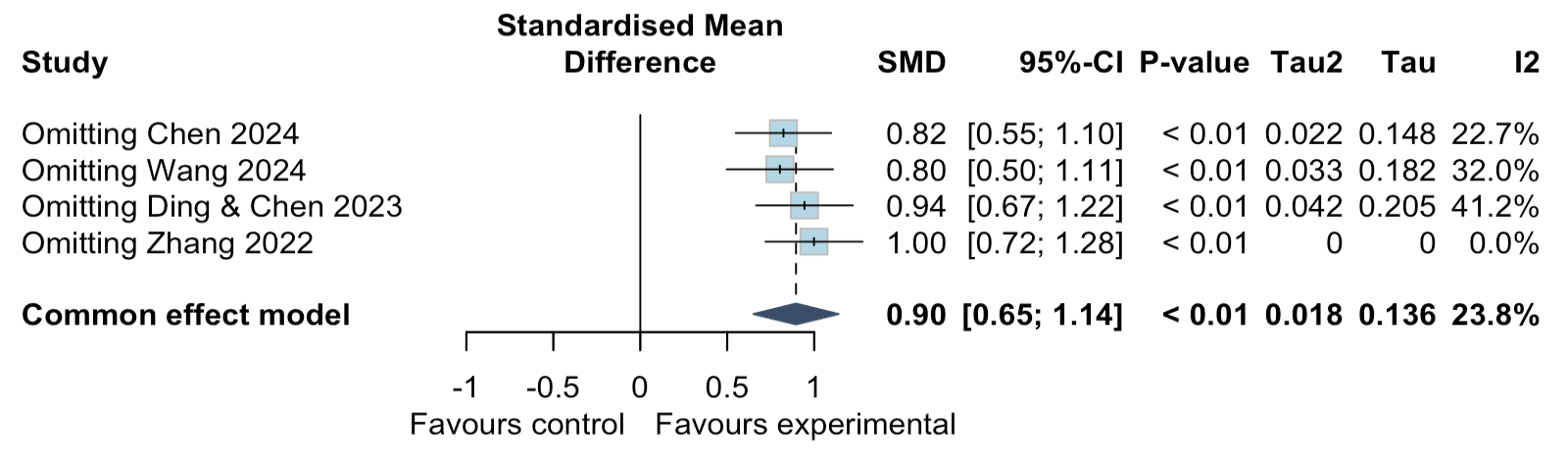

E


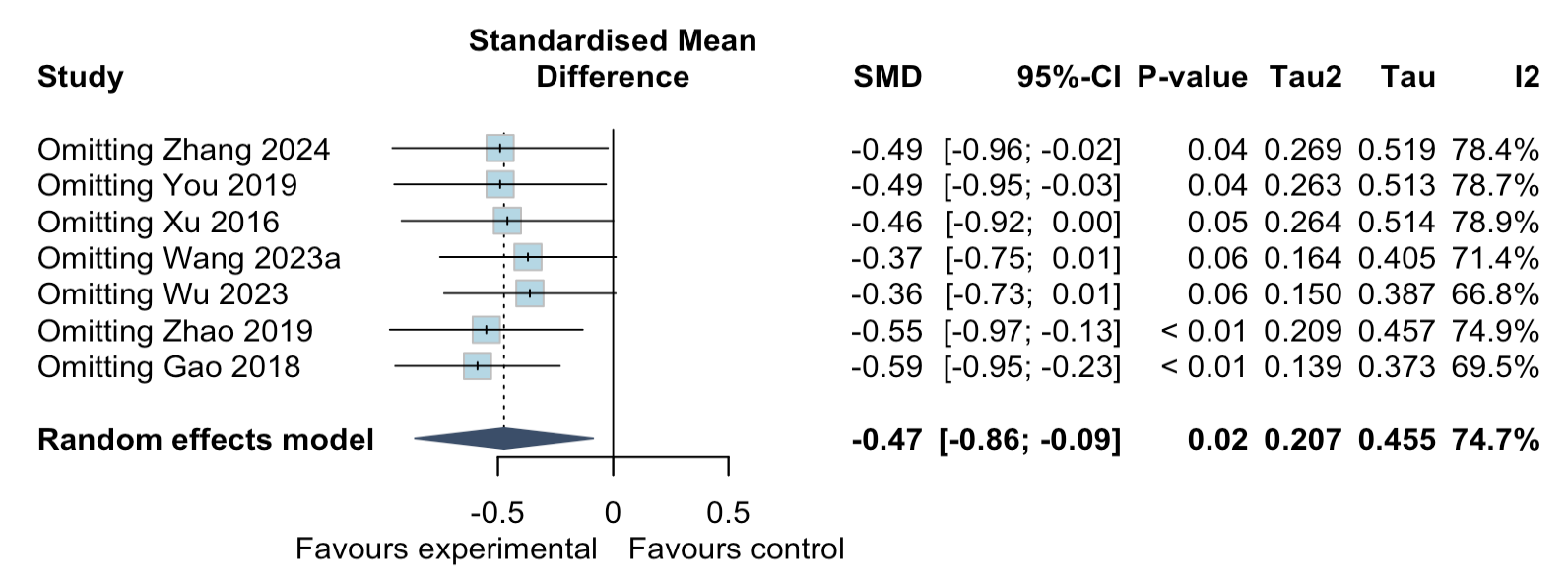

F


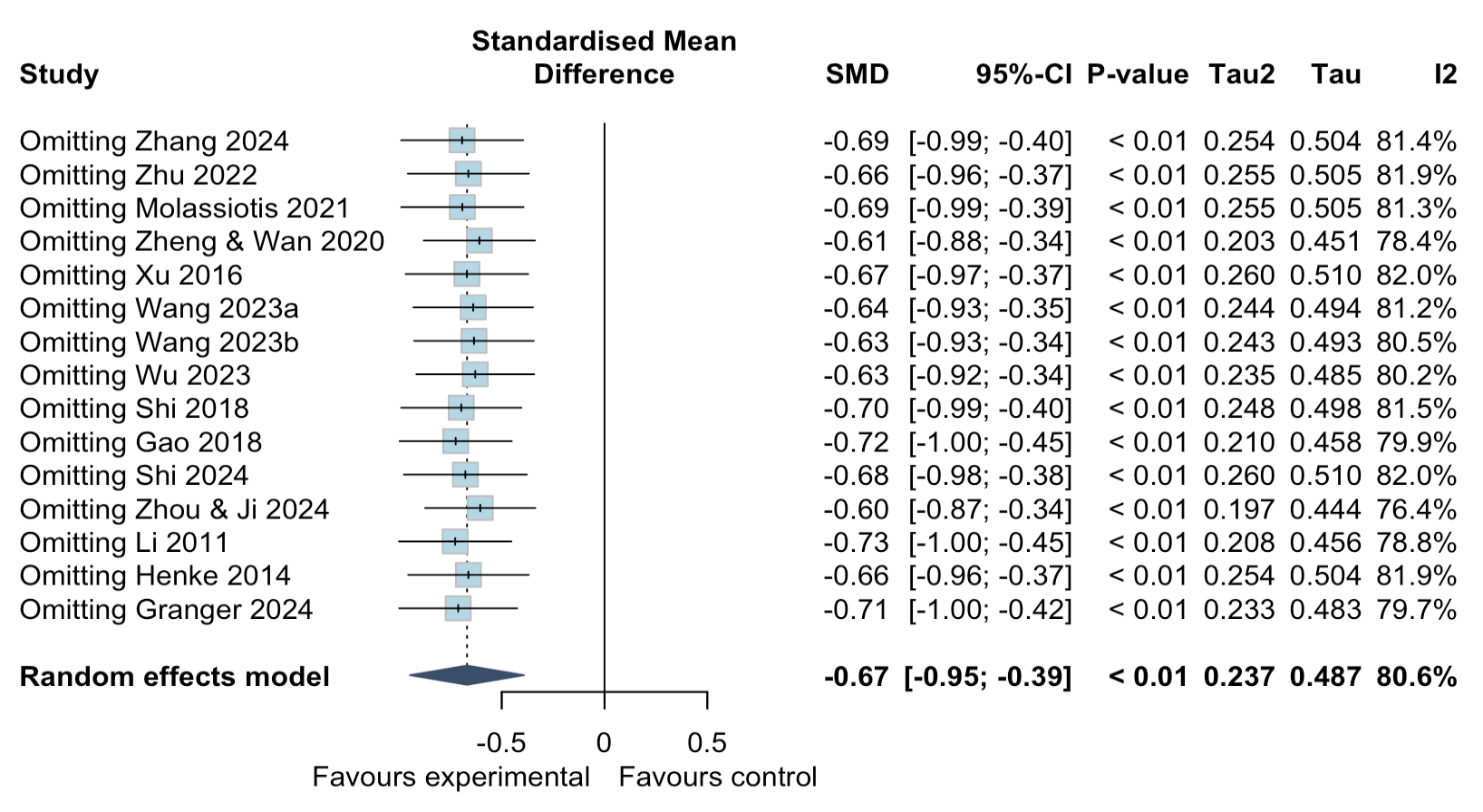

G


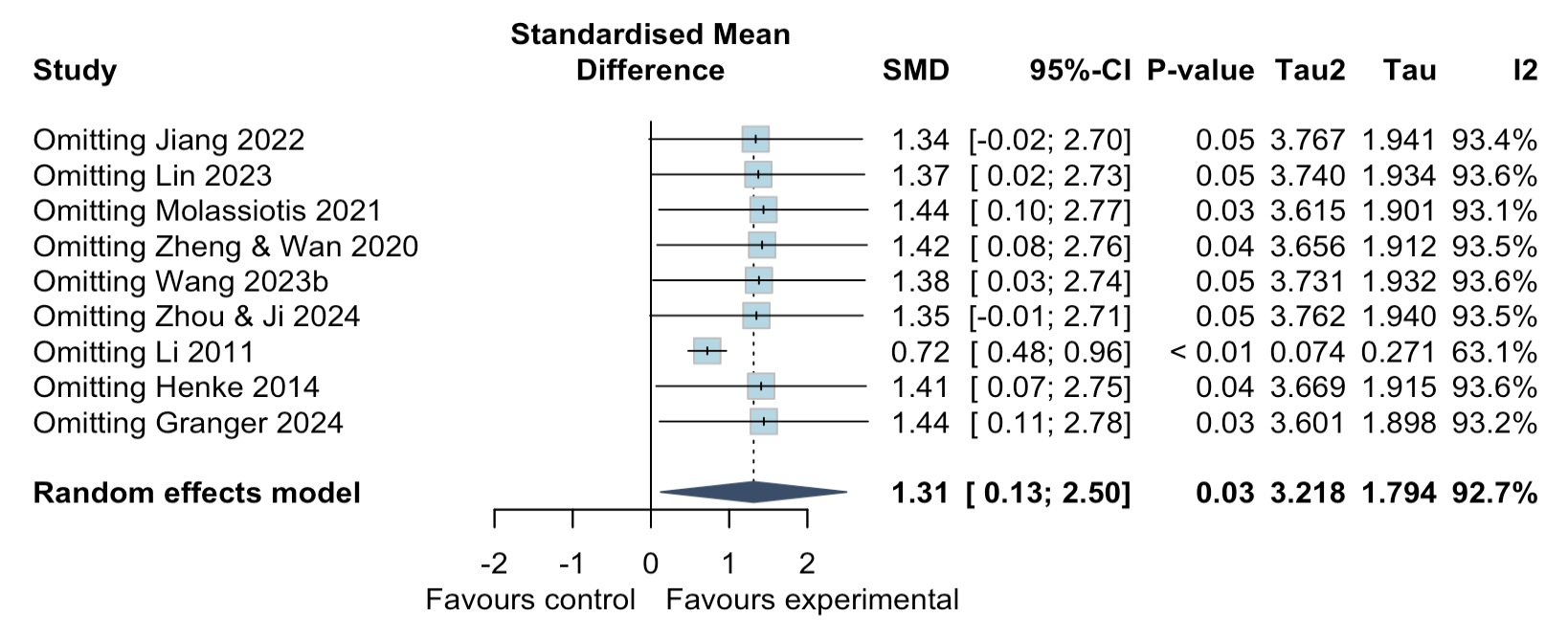


Figure S2. Funnel plots. A) cough severity; B) cough severity (after trim and fill analysis); C) dyspnea

A


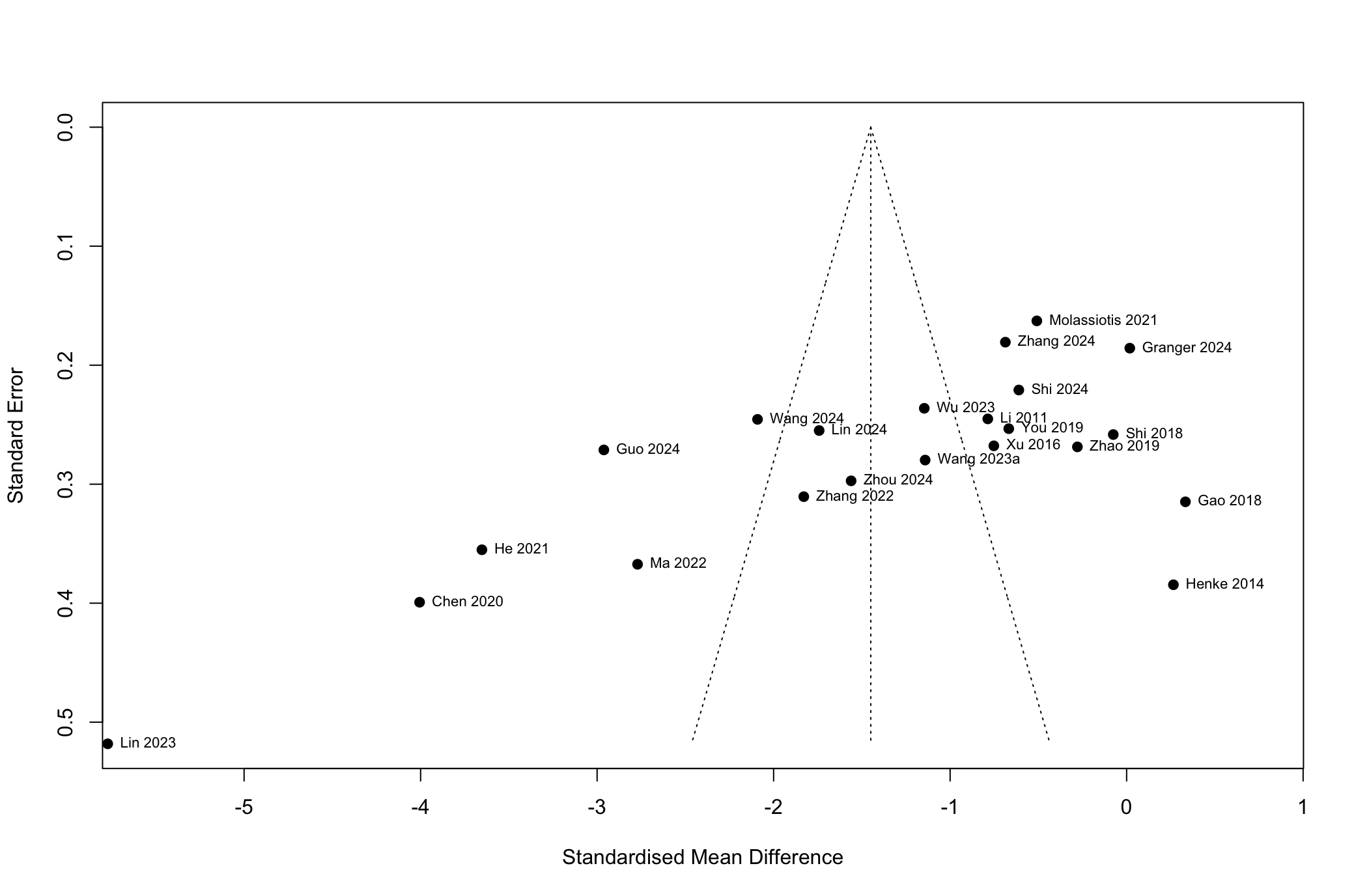


B


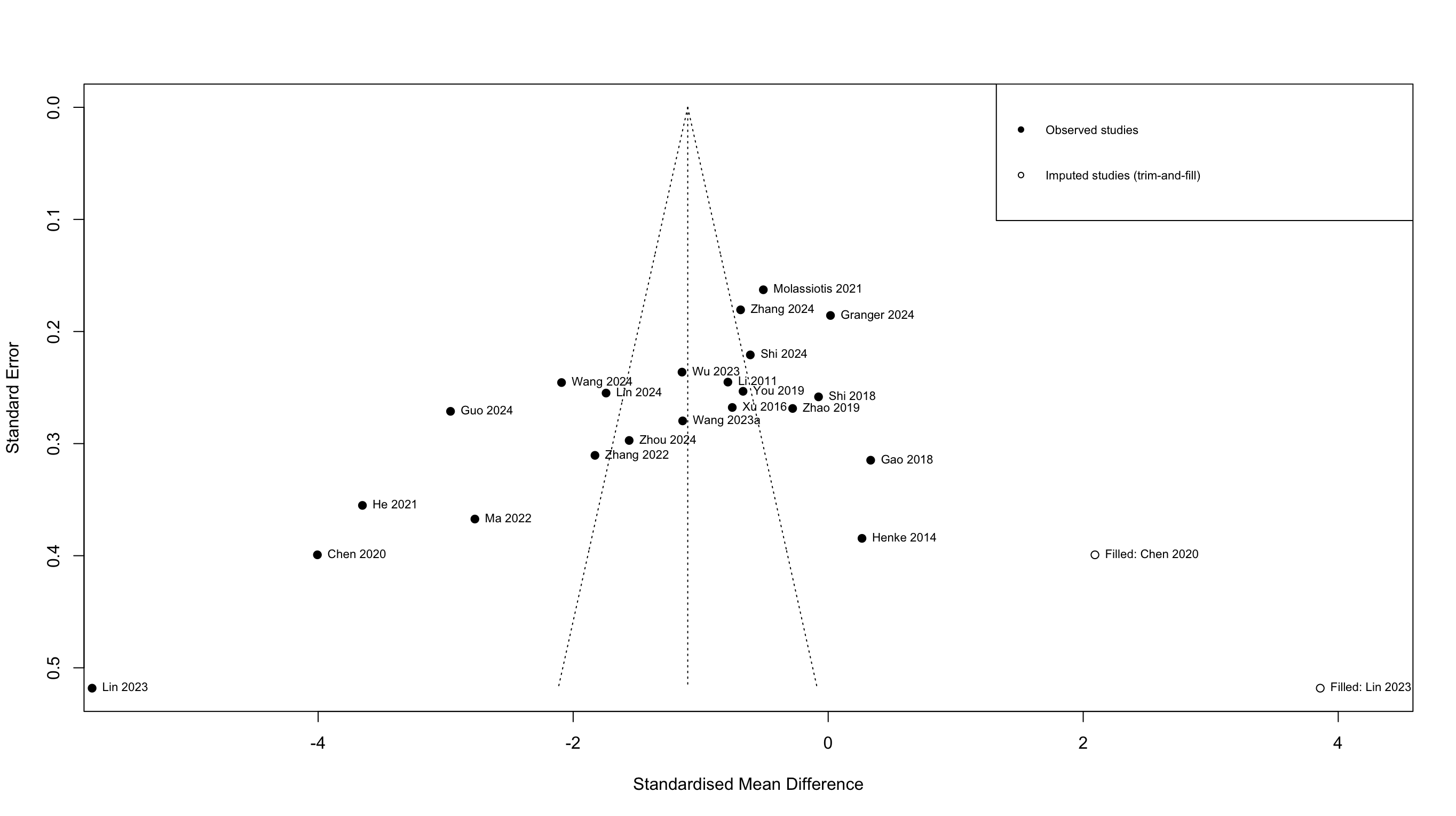


C


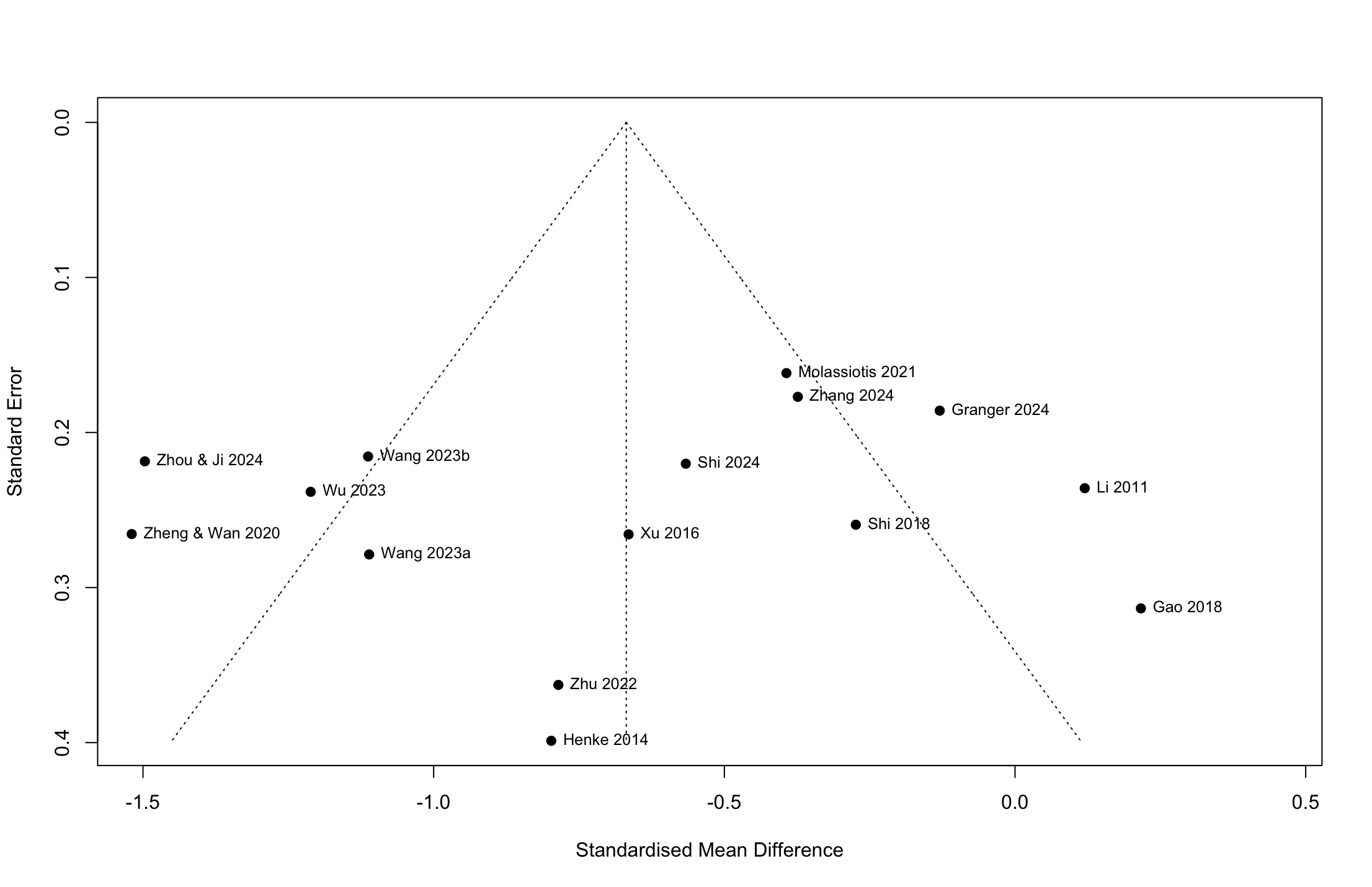

Supplement: Supplementary file 1 — Table S1: Search strategy. Table S2: GRADE assessment. Figure S1: Sensitivity analyses. (A) cough severity (no CSS); (B) cough severity (CSS‐D); (C) cough severity (CSS‐N); (D) cough‐related quality of life; (E) expectoration; (F) dyspnea; (G) general quality of life. CI, confidence interval; CSS, cough symptom score; CSS‐D, cough symptom score‐daytime; CSS‐N, cough symptom score‐nighttime; SD, standard deviation; SMD, standardised mean difference. Figure S2: Funnel plots. (A) cough severity; (B) cough severity (after trim and fill analysis); (C) dyspnea. [file JOCN-35-3335-s001.docx]
